# GPseudoRank: a permutation sampler for single cell orderings

**DOI:** 10.1093/bioinformatics/bty664

**Published:** 2018-07-25

**Authors:** Magdalena E Strauß, John E Reid, Lorenz Wernisch

**Affiliations:** 1 MRC Biostatistics Unit, School of Clinical Medicine, University of Cambridge, Cambridge, UK; 2 Alan Turing Institute, London, UK

## Abstract

**Motivation:**

A number of pseudotime methods have provided point estimates of the ordering of cells for scRNA-seq data. A still limited number of methods also model the uncertainty of the pseudotime estimate. However, there is still a need for a method to sample from complicated and multi-modal distributions of orders, and to estimate changes in the amount of the uncertainty of the order during the course of a biological development, as this can support the selection of suitable cells for the clustering of genes or for network inference.

**Results:**

In applications to scRNA-seq data we demonstrate the potential of GPseudoRank to sample from complex and multi-modal posterior distributions and to identify phases of lower and higher pseudotime uncertainty during a biological process. GPseudoRank also correctly identifies cells precocious in their antiviral response and links uncertainty in the ordering to metastable states. A variant of the method extends the advantages of Bayesian modelling and MCMC to large droplet-based scRNA-seq datasets.

**Availability and implementation:**

Our method is available on github: https://github.com/magStra/GPseudoRank.

**Supplementary information:**

Supplementary data are available at *Bioinformatics* online.

## 1 Introduction

Single cell RNA-seq (scRNA-seq) technology can assay mRNA expression levels in individual cells and has revealed substantial inter-cell heterogeneity. Technical noise contributes to this heterogeneity but part of it is attributable to biologically meaningful inter-cell differences, see, for instance, Brennecke *et al.* (2013); Vallejos *et al.* (2015). Due to the destruction of the cells as a result of the measurement process, scRNA-seq only provides a single measurement per cell (Stegle *et al.*, 2015), never time series data following the development of the same single cell. However, individual cells progress through changes at different time scales (Trapnell *et al.*, 2014). Thus, it is possible to obtain a form of time series data even from cross-sectional data by statistical means, an approach referred to as pseudotime ordering.

Most approaches to pseudotemporal ordering are based on representing cells as *n_g_*-dimensional vectors, where *n_g_* is a selected number of genes in a cell. Algorithms exploit the neighborhood structure of these vectors to find a pseudotemporal ordering, a linear ordering of all or most cells so that cells which are close in $R^{n_{g}}$ are also close in the linear ordering.

Wanderlust (Bendall *et al.*, 2014) and SLICER (Welch, 2017; Welch *et al.*, 2016) are two examples of methods based on *k* nearest neighbors graphs. SLICER additionally first applies LLE (local linear embedding) (Roweis and Saul, 2000) for dimensionality reduction. A number of methods are based on diffusion maps (Angerer *et al.*, 2016; Haghverdi *et al.*, 2015, 2016; Setty *et al.*, 2016). TSCAN (Ji and Ji, 2016, 2015) is based on the construction of a minimum spanning tree (MST) between centroids of clusters, with an intermediate clustering step. Another well-known method using MST and clustering is Monocle 2 (Qiu *et al.*, 2017), which applies graph structure learning (Mao *et al.*, 2015).

The approaches mentioned above and a number of others provide singular pseudotime orderings without modelling uncertainty. Campbell and Yau (2016) examined the stability of Monocle’s pseudotime estimation when applied to random subsets of cells. They showed that the estimates can vary significantly. Thus, quantification of uncertainty in pseudotime is crucial to avoid overconfidence. There are two existing methods for pseudotime estimation using MCMC to sample from a posterior distribution (Campbell and Yau, 2016; Reid and Wernisch, 2016), and a few others using variational methods (Ahmed *et al.*, 2018; Reid and Wernisch, 2016; Welch *et al.*, 2017). Both use Gaussian processes (GPs, see Section 2.1) to model the data. However, these methods sample from, or approximate in the case of variational inference, posterior distributions of continuous pseudotime vectors in $R^{n}$, rather than sampling the ordering as a permutation.

We propose GPseudoRank, an algorithm sampling from a posterior distribution of pseudo-orders instead of pseudotimes, avoiding the exploration of pseudotime assignments that all map to the same ordering. MCMC samplers [such as NUTS (Hoffman and Gelman, 2014)] suitable for use in continuous pseudotime spaces make local moves that can have problems exploring bi-modal posteriors. GPseudoRank, by contrast, exploits a range of local and long-distance MCMC moves tailored to traverse the space of permutations efficiently. It also provides continuous pseudotime estimates by deriving a pseudotime vector from a fixed ordering through a deterministic transformation. This is based on the observation that most continuous pseudotime vectors with high likelihood are concentrated around pseudotime vectors derived from orderings through this transformation.

## 2 Materials and methods

### 2.1 Single-cell trajectories as stochastic processes

We assume we have preprocessed log-transformed gene expression data in the form $y_{g}(c)$ of gene $g=1,\ldots ,n_{g}$, in cell c=1,…,T (see Section 2.7 for preprocessing steps). We start with a vector of time points $\tau =(\tau_{1},\ldots ,\tau_{T})$ and define an ordering of cells as a permutation $o=(o_{1},\ldots ,o_{T}),\;o_{i}\in \{1,\ldots ,T\},\;o_{i}\neq o_{j}$ for i≠j, where *o_i_* is the index of the cell assigned to time *τ_i_* in the ordering. We model the gene expression trajectories $y_{g}=(y_{g}(o_{1}),\ldots ,y_{g}(o_{T}))$ for each gene *g* by Gaussian processes (GPs) (Rasmussen and Williams, 2006), conditional on an ordering **o** of the cells. A GP is a distribution over functions of time in terms of a mean function *μ* and a covariance function Σ. For an input vector $\tau =(\tau_{1},\ldots ,\tau_{T})$ of time points, μ(τ) is a vector of *T* mean values of function evaluations at these time points and Σ(τ) a *T *×* T* matrix of covariances of function evaluations at these points. The distribution of functions f∼GP(μ,Σ) is described by stating that, for any vector of time points $\tau =(\tau_{1},\ldots ,\tau_{T})$, evaluations $f(\tau_{i})$ follow a multivariate normal $\left(f(\tau_{1}),\ldots ,f(\tau_{T}))∼N_{T}(\mu (\tau ),\Sigma (\tau )\right)$. Here we use a squared exponential covariance function for Σ:

(1) $${\left[\Sigma (\tau ,\sigma_{w}^{2},l,\sigma_{\epsilon }^{2})\right]}_{i,j}=\sigma_{w}^{2}\;\text{exp}\;\left(-\frac{{\left(\tau_{j}-\tau_{i}\right)}^{2}}{2l^{2}}\right)+\delta_{ij}\;\sigma_{\epsilon }^{2}$$

where $\sigma_{w}^{2}$ is a scale parameter, *l* a length scale and $\sigma_{\epsilon }^{2}$ a term representing measurement noise.

Given an ordering **o**, the expression data for gene *g* can be ordered accordingly: $y_{g}(o)=(y_{g}(o_{1}),\ldots ,y_{g}(o_{T}))$ and we model this trajectory as

(2) $$y_{g}(o)∼N_{T}(\mu (\tau ),\Sigma (\tau ,\sigma_{w}^{2},l,\sigma_{\epsilon }^{2}))$$

for each gene $g=1,\ldots ,n_{g}$, where $\tau =(\tau_{1},\ldots ,\tau_{T})$ are time points. In practice, we assume a zero-mean GP: μ=0. To adjust the data accordingly we subtract the overall mean across all genes and cells from each entry in the matrix of gene expression levels (see Section 2.7.2).

### 2.2 Geodesic mapping

Pseudotime should not be confused with physical time in which cell development unfolds. In order to identify the latent time points $\tau =(\tau_{1},\ldots ,\tau_{T})$, which we assume to be unknown, together with the smoothness parameters of the GP, we have to make additional assumptions. The overall scale can be fixed by assuming $\tau_{i}\in [0,1]$. Each cell can then be assigned some *rank time* from equidistant time points ((i−0.5)/T|i=1,…,T). Rank time is similar to the concept of master time developed in Welch *et al.* (2017). Simply identifying pseudotime with rank time has some drawbacks. Rank time depends on the number of cells sampled per capture time, which often is rather arbitrary. It also does not allow any local change in scale. We therefore suggest a different route for identifying latent time points. We assume the covariance structure, essentially the smoothness of the process, is independent of time, that is, the GP is *stationary*. Pseudotime can then be considered a latent variable measuring biological development rather than physical time (Ahmed *et al.*, 2018; Campbell and Yau, 2016; Reid and Wernisch, 2016; Welch *et al.*, 2017). For periods of slower development, for example, pseudotime intervals will be shorter than physical time intervals and longer for faster development. In order to account for such change in scale over time we compute time points for any given ordering **o** as follows (recall *o_j_* is the index of the cell in position *j*).

(3) $$\begin{array}{c} {\tilde{\tau }}_{1}(o)=0,\\ {\tilde{\tau }}_{j+1}(o)={\tilde{\tau }}_{j}(o)+||y(o_{j}),y(o_{j+1})|{|}_{2},\;j=1,\ldots ,T-1 \end{array}$$

where $y(o_{j})={\left(y_{1}(o_{j}),\ldots ,y_{n_{g}}(o_{j})\right)}^{T}$ and $||.|{|}_{2}$ is the Euclidean norm in $R^{n_{g}}$. We set $\tau (o)=\tilde{\tau }(o)/\max(\tilde{\tau }(o))$ to obtain pseudotimes τ(o) in the interval [0, 1]. For cells next to each other in the order **o**, this mapping puts them closer in pseudotime if they are similar in their expression profiles and further apart if they are less so. That is, the *j*-th time point *τ_j_* is the geodesic distance of cell *o_j_* from the first cell *o*_1_, where we approximate the geodesic distance as the sum of the Euclidean distances between the cells ranked next to each other, similar to the dimensionality reduction method Isomap (Tenenbaum *et al.*, 2000). Geodesic distances have previously been used for pseudotime estimation, see for instance Qiu *et al.* (2017); Welch (2017).

### 2.3 Gaussian process priors

The correct ordering **o** of cells is distinguished by comparatively low measurement noise $\sigma_{\epsilon }^{2}$ in (1), since most of the variation is captured by the trajectory whose variability is determined by the scale parameter $\sigma_{w}^{2}$. Therefore, informative priors for the noise parameters are necessary to ensure the model concentrates probability mass around the correct order and to avoid that a sampling or estimation algorithm gets trapped in local modes. Furthermore, since total variability is a sum of measurement noise and signal variability, we sample only $\sigma_{w}^{2}$ and set $\sigma_{\epsilon }^{2}=V-\sigma_{w}^{2}$, where *V* is the sample variance taken across the entire $n_{g}\times T$ matrix of gene expression levels of *T* cells for *n_g_* genes. The priors are as follows:

$$\text{log}(\sigma_{w})∼N(\text{log}(\sqrt{0.9\cdot V}),0.01)$$

$$\text{log}(l)∼N(\text{log}(\frac{1}{2}),v)$$

o∼uniform(permutations of {1,…,T})

$$y_{g}(o)|\sigma_{w}^{2},l∼N_{T}(0,\Sigma (\tau (o),\sigma_{w}^{2},l,V-\sigma_{w}^{2}))$$

We set *v *=* *0.01 for all the single-cell datasets considered (see Section 2.7.2). A strong prior is preferable for single-cell data because of their high noise levels. With a vague prior on the length scale, the posterior tends to be too short and the GP tends to overfit.

### 2.4 MCMC sampling

Markov Chain Monte Carlo (MCMC) methods (Gilks *et al.*, 1996) are widely used to sample from continuous posterior densities in Bayesian statistics. After convergence, MCMC chains provide samples from the posterior distribution. More specifically, our method uses a Metropolis-Hastings approach (Hastings, 1970; Metropolis *et al.*, 1953). For each given state of the Markov Chain, a new state is proposed using a proposal distribution, and accepted with a probability given by an acceptance ratio. While the construction of proposal distributions is often straightforward in the continuous case, we developed a set of proposal moves to sample from the discrete distribution of orders (see Section 2.5). For the sampling of the GP parameters, we use Gaussian proposal distributions, adapting their standard deviation during burn-in aiming at acceptance rates between 0.45 and 0.5.

### 2.5 Sampling orderings

In the following, we propose a Metropolis-Hastings algorithm for the sampling of the orderings. Preliminary experience with a variety of combinatorial moves to sample permutations led to the following set of five core moves, each with probability *p_j_*, j=1,…,5. In the following, we use sampling parameters $n_{0},\gamma ,n_{3},n_{3a}$:

1. Move 1, **iterated swapping of neighboring cells**: draw the number of swaps to be applied, *r*_1_, uniformly from $1,\ldots ,n_{0}$ and draw *r*_1_ swap positions $P_{1},\ldots ,P_{r_{1}}$ from 1,…,T−1 with replacement. Then iterate for $j=1,\ldots ,r_{1}$: swap cell at position *P_j_* with its neighbor at position $P_{j}+1$.
2. Move 2, **swapping of cells with short *L*^1^-distances**: select two positions *i* and *j* according to probability $p_{ij}\propto \;\text{exp}(-d{\left(c_{i},c_{j}\right)}^{2}/\gamma )$, where *d* refers to the *L*^1^ distances of cells *c_i_* and *c_j_* (as *n_g_*-dimensional vectors) in these positions. Move *c_i_* to position *j* and *c_j_* to position *i*.
3. Move 3, **reversing segments between cells with short *L*^1^-distances**: obtain two positions *i* and *j* as in move 2 and reverse the ordering of all cells in between, including cells at *i* and *j*.
4. Move 4, **short random permutations**: draw a number *r*_2_ of short permutations uniformly from $1,\ldots ,n_{3}$. For each $j=1,\ldots ,r_{2}$, draw a number $r_{3,j}$ uniformly from $3,\ldots ,\max(n_{3a},3))$ and a cell position *k_j_* uniformly from $1,\ldots ,T-r_{3,j}$. Randomly permute the cells at positions $k_{j},\ldots ,k_{j}+r_{3,j}$.
5. Move 5, **reversing the entire ordering**.

The rationale for moves 2 and 3 is that two cells which are positioned apart in the ordering should only be exchanged (move 2) or the segment between them reversed (move 3) if these cells have similar expression profiles and the smoothness of the trajectory remains intact after the move. For move 1, we use a default setting of $n_{0}=\left\lfloor T/4\right\rfloor$ for the simulation studies. For move 4, we set $n_{3}=\left\lfloor T/20\right\rfloor$, and $n_{3a}=\left\lfloor T/12\right\rfloor$. The distributions for choosing moves 2 and 3 may be tempered, that is taken to the power of a factor 0<α<1, to lower acceptance rates if required.

For the simulation studies, we apply all possible combinations of moves 1 to 4 with equal probabilities and move 5 with a probability of 0.002. For the five experimental datasets analyzed (see Section 2.7.2), we chose default parameters depending on the number of cells (see Section 6 of the Supplementary Material), and slightly adapted some of them to optimize convergence rates. For details on the parameters for the proposal distribution for all the datasets, see Table 5 in the Supplementary Material.

As our posterior distribution is a symmetric function of the order, each order and its reverse will be sampled with equal probability from the posterior distribution. We remove this symmetry in further analysis by reversing orders, which are negatively correlated with the capture times, if available, or else with marker genes and invert posterior orders accordingly. For details and an application, see Section 4 of the Supplementary Material.

### 2.6 Method for large datasets

To decrease run times for very large datasets, we perform a preprocessing step clustering cells into a large number of very small clusters using k-means clustering. If capture times are provided this is done separately for each group of cells at the different capture times. The recommended number of clusters for each capture time is 1/8th of the number of cells at the capture time. One might also want to set an absolute minimum number of cells per capture time. The number of clusters may be decreased substantially for very large datasets, as they include larger numbers of similar cells, making our method applicable to datasets with tens of thousands of cells. We then apply GPseudoRank to the *k* centroids, reducing the computational complexity of each individual likelihood computation. The proposed preprocessing step also drastically reduces the number of samples required for convergence by reducing the size of the sample space.

The posterior distribution of orderings of the centroids of the mini-clusters is obtained. To the individual cells of a mini-cluster, we assign the posterior pseudotimes of its centroid. To assess the accuracy of this approximation, we applied it to two medium-sized datasets with 307 and 550 cells respectively, where inference with the exact model is feasible. For details and a comparison to sparse GP approximation, see Section 3 of the Supplementary Material.

### 2.7 Datasets

#### 2.7.1 Simulated data

The efficacy of the individual moves and of combinations of different moves for different types of data is first assessed on simulated data. We simulate *n_g_* = 50 genes for *T *=* *90 cells. For each simulation study, we generate 16 datasets. On each of these datasets, we run MCMC chains using all the possible combinations of the four proposed moves (with equal probability for combinations of more than one move). Since in the simulations we are mostly interested in the assessment of ordering moves and not any parameter estimation, we fix them to their true values and fix time points to rank time.

**Simulation 1: three capture times, low noise**. Each of the 16 datasets is generated as follows. First 90 temporal input points are drawn uniformly from [0, 1]. Then for each of the 50 genes in each of the simulated datasets, a parameter set for a GP underlying the trajectory of the simulated gene is drawn from

$$\text{log}(\sigma_{w})∼N(0,0.1)$$

log(l)∼N(log(0.4),0.1)

$$\text{log}(\sigma_{\epsilon })∼N(\text{log}(1/\sqrt{2}),0.1).$$

The data are assumed to be obtained at three capture times with 30 cells each.

**Simulation 2: two capture times, low noise**. The setup is similar to simulation 1, but with two capture times, where 30 cells are assigned to the first capture time, and the remaining 60 to the second.

**Simulation 3: three capture times, high noise**. The setup is similar to simulation 1, but $\text{log}(\sigma_{\epsilon })∼N(0,0.1)$.

#### 2.7.2 Single cell RNA-seq and RT-PCR datasets

Shalek *et al.* (2014) examined the response of primary mouse bone-marrow-derived dendritic cells in three different conditions using single-cell RNA-seq. We apply GPseudoRank to the lipopolysaccharide stimulated (LPS) condition. Shalek *et al.* (2014) identified four modules of genes. As in Reid and Wernisch (2016), we use a total of 74 genes from the four modules with the highest temporal variance relative to their noise levels. The number of cells is 307, with 49 unstimulated cells, 75 captured after 1 h, 65 after 2 h, 60 after 4 h, and 58 after 6 h. We use an adjustment for cell size developed by Anders and Huber (2010), also used in Reid and Wernisch (2016).

Klein *et al.* (2015) generated a droplet-based dataset of mouse embryonic stem cells after Leukemia inhibition factor withdrawal (0d, 2d, 4d, and 7d). We apply GPseudoRank to the main branch with 1543 cells identified in a previous publication (the third branch in Fig. 2c of Haghverdi *et al.*, 2016). Shin *et al.* (2015) generated an *in vivo* scRNA-seq dataset of mouse adult hippocampal quiescent neural stem cells and their immediate progeny (Shin *et al.*, 2015) and used 101 cells for their subsequent analysis. Stumpf *et al.* (2017) generated RT-PCR data following the development of mouse embryonic stem cells along the neuronal lineage (0 h, 24 h, 48 h, 72 h, 96 h, 120 h, and 172 h) (550 cells after preprocessing). Shalek *et al.* (2013) obtained scRNA-seq data from mouse bone-marrow-derived dendritic cells after exposure to lipopolysaccharide for 4 hours (18 cells). We refer to the datasets as Shalek, Klein, Shin, Stumpf, and Shalek13, respectively. For a description of the datasets, their availability (all publicly available) and preprocessing steps, see Section 2 of the Supplementary Material. For all datasets with different capture times (excepting the Stumpf dataset, which only contains 96 genes), we use an ANOVA test (Murphy, 2012, ch. 7) for differences of mean expression (the mean being taken across one individual capture time) for different capture times to filter a set of genes most relevant to the ordering. In the absence of capture times, we use genes with both a high mean expression and high variance (for details see Section 2 of the Supplementary Material).

**Fig. 1.**
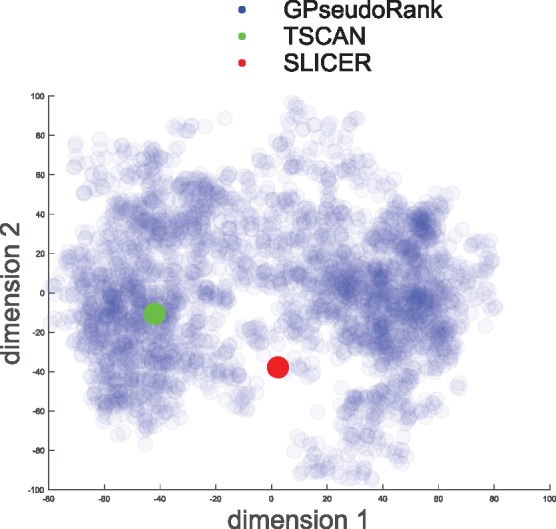
Shalek data. Multi-dimensional scaling. Distribution sampled with GPseudoRank, point estimates with TSCAN and SLICER. Each dot corresponds to one vector of cell positions from the posterior distribution. Semi-transparency of the points allows identification of ares of higher density, that is local modes of the posterior distribution

### 2.8 Convergence assessment and analysis of posterior distribution

For thorough convergence assessment, we run multiple MCMC chains. For the simulated datasets, we run 100 000 iterations per MCMC chain for five chains and apply a thinning factor of 10. For the scRNA-seq datasets, we used the same thinning factor and at least three MCMC chains for convergence analysis (12 for those datasets (Shalek and Shin) where we analyzed datasets without capture times). The number of samples required depends on the dataset (see Table 5 in the Supplementary Material with all the examples, providing approximate guidance on the number of samples required for similar datasets). In order to assess convergence and not to bias the sampler towards specific orderings, all chains are seeded with random starting orders and with random GP parameters sampled from the prior distribution. However, we do restrict starting orders to permutations of cells within, but not across capture times. The restriction, while resulting in faster convergence, is not actually necessary (see Section 4 of the Supplementary Material for details).

To check convergence, we use the Gelman-Rubin $\hat{R}$-statistic (Gelman and Rubin, 1992), corrected for sampling variability (Brooks and Gelman, 1998), implemented in the R-package coda (Plummer *et al.*, 2006). The $\hat{R}$-statistic estimates the factor by which the pooled variance across all the chains is larger than the within-sample variance. For convergent chains, $\hat{R}$ approaches 1 as the number of samples tends to infinity. According to Brooks and Gelman (1998), convergence may be assumed to have been reached if $\hat{R}<1.2$. We apply the stricter recommendation of $\hat{R}<1.1$ (Gelman and Shirley, 2011). We compute the $\hat{R}$ statistics for the following two quantities: first, the log-likelihood, and second the *L*^1^-distances of the sampled cell positions from a fixed reference set of cell positions, for which we use the true order, if known, and 1,…,T, where *T* is the number of cells, in case of scRNA-seq data. We compute the $\hat{R}$ statistics a number of times during sampling, each time discarding the first 50% (Gelman and Shirley, 2011). We compare the speed of convergence for different combinations of proposal moves in the simulation studies. See Section 1 in the Supplementary Material for details.

While distance from reference orders is an efficient way of obtaining a statistic for convergence assessment, more insights into the structure of the orders can be obtained from low dimensional representations, for example by MDS (multidimensional scaling) (Borg and Groenen, 2005) on the (Euclidean) distance matrix of the position vectors of the cells. It also allows us to visualize comparisons with alternative methods. Figure 1 shows the TSCAN solution located in one of the areas of higher density of the GPseudoRank solution, while the solution found by SLICER lies somewhat in between two modes, around the center of the distribution.

## 3 Results

### 3.1 Simulation studies

This section summarizes the insights gained from the simulation studies. For details on the assessment criteria and results, see Section 1 in the Supplementary Material.

**Simulation 1**. Any combination of moves leads to good convergence, and although there are differences in the speed and level of convergence, any combination of moves is recommended.

**Simulation 2**. There are only two capture times, hence there is more variety in the starting orders for each chain. The performance of the combinations of moves is different from simulation 1. Move 3 performs better than any other single move.

Move 3 generally traverses the space of permutations faster by reversing whole segments of an ordering and it is the only move for which all $\hat{R}$-statistics go below 1.1 within the first 10 000 thinned samples. The combination of moves ranked first according to the criteria described in Section 1 of the Supplementary Material is the combination 1, 2, 3, and 4 of all the moves.

**Simulation 3**. All moves and combinations thereof perform well in this situation, though move 3, while still achieving reasonable levels of convergence, is now the comparatively less well performing single move. The combination of all four moves performs well.

### 3.2 Pseudotemporal uncertainty varies during response to infection

**Fig. 2.**
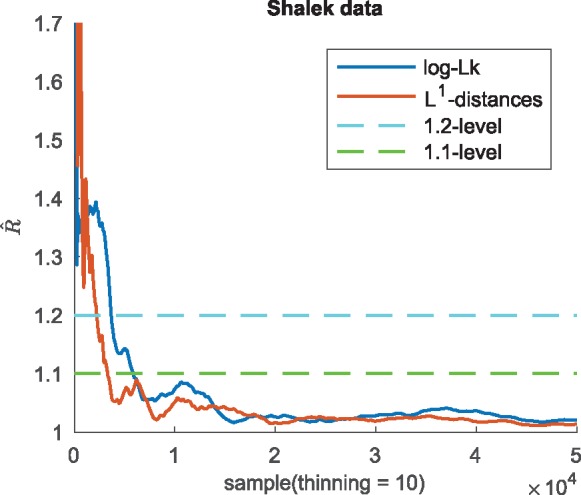
Convergence analysis for GPseudoRank. Gelman-Rubin statistics for the log-likelihood and for the *L*^1^-distances of the sampled permutations of cell positions from the reference permutation (Shalek data)

For the scRNA-seq data from Shalek *et al.* (2014), collected at five different capture times, the true cell ordering is unknown. To check convergence of orders the $\hat{R}$-statistic is computed both on the log-likelihood and on the *L*^1^-distances of the permutation of cell positions to an arbitrary reference permutation (Fig. 2).

Figure 2 shows that a threshold for the $\hat{R}$-statistic of 1.1 has been reached after 6000 thinned samples (see also Table 5 in the Supplementary Material). We therefore discard a burn-in of 3000 thinned samples at the beginning of each chain, as recommended by Gelman and Shirley (2011). Indeed, by the 1.1 threshold for the $\hat{R}$ statistic 6000 thinned samples would have been sufficient for convergence.

**Fig. 3.**
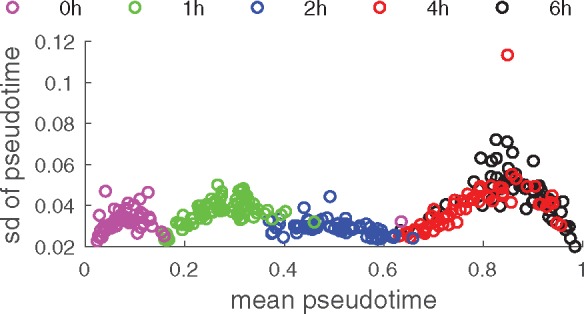
Uncertainty of pseudotime as a function of mean pseudotime. For each cell, the mean pseudotime is plotted along the x-axis, and the respective standard deviation along the y-axis. Cells are colored by capture time

Figure 1 demonstrates again the value of providing a posterior distribution for orders, rather than a single estimate: TSCAN and SLICER give different results. Figure 3 illustrates the uncertainty of the pseudotime over the mean pseudotime. To ensure that the inverted U-shape in the amount of uncertainties of the first two capture times at 0 h and 1 h is not a sampling artifact, cells from these capture times were mixed together for initializing the sampler (that is, capture time information was discarded). On the other hand, despite being separated during initialization of the sampler, cells from capture times 4 h and 6 h are completely merged, again indicating that the sampler has reached convergence.

Overall uncertainty in the ordering of cells is markedly lower around capture time 2 h, when the reaction to the infection has set in, but is not yet complete. The slight U-shape in the amount of uncertainty for capture times 0 h, 1 h, and 4h/6h seems to be an experimental batch effect of capturing multiple heterogeneous cells at different time points. Within a batch (or merged batches 4 h and 6h) cells which are either lagging behind or slightly ahead in their development are assigned a more specific pseudotime with lower uncertainty behind or ahead of the bulk of cells whose pseudotimes are, in contrast, more interchangeable with higher uncertainty. However, for other datasets (for instance with different time intervals between capture times) this graph might look different, as, for example, in Supplementary Figure 7 for the Stumpf dataset.

**Fig. 4.**
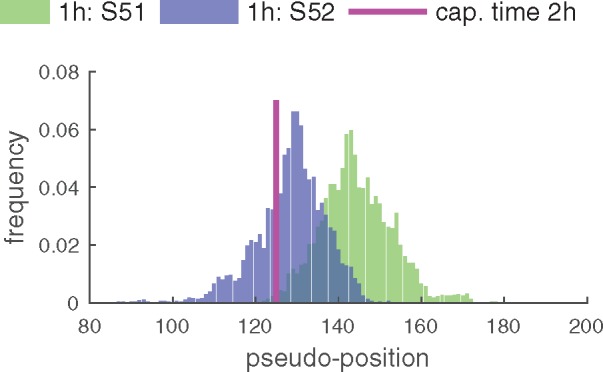
Posterior distribution of cell positions of the precocious cells. For each posterior position of the cells we plot the frequency at which this position occurs among all samples. One random MCMC chain was used. Both of the precocious cells have a high probability of being located within capture time 2 h, with S51 likely to be ahead of S52

**Fig. 5.**
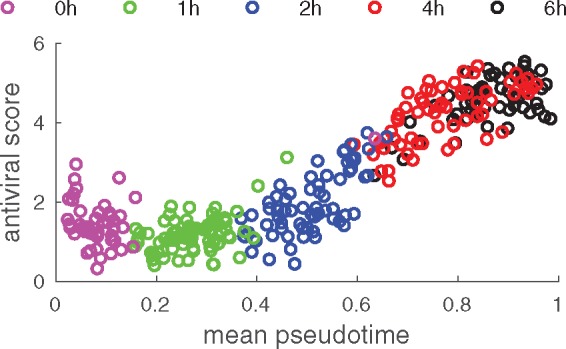
Core antiviral score as a function of mean pseudotime

GPseudoRank identifies two precocious cells, pointed out in the original analysis by Shalek *et al.* (2014), ahead in terms of their response to the stimulus, see Figure 4. Shalek *et al.* (2014) identified a set of genes particularly associated with antiviral response. Ahmed *et al.*, 2018 and Reid and Wernisch (2016) also used this score to demonstrate that their methods identify two cells at capture time 1 h precocious in their antiviral response. Figure 5 shows the average expression of a set of genes associated with antiviral response for each cell. As expected, this antiviral score increases over pseudotime, confirming that the pseudotime assignment captures a biological phenomenon. In contrast to Figure 5, both DeLorean (Reid and Wernisch, 2016) and GrandPrix (Ahmed *et al.*, 2018) show considerable edge effects in comparable plots (Reid and Wernisch, 2016, Fig. 4, Ahmed *et al.*, 2018, Fig. 2). Such edge effects are not biologically motivated and presumably algorithmic artifacts which GPseudoRank is able to avoid by restricting pseudotimes to a finite interval and by using a geodesic mapping.

### 3.3 Posterior uncertainty modelling for droplet-based scRNA-seq

**Fig. 6.**
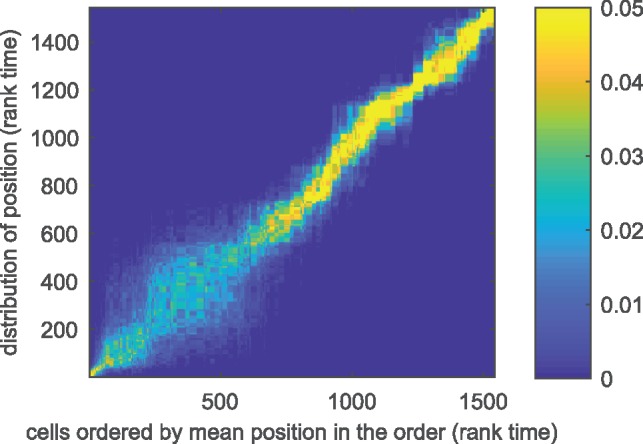
Klein data. Posterior distribution of cell positions. Posterior probabilities of the positions of the cells in terms of rank time: the mean cell position is along the x-axis, the posterior density is plotted along the y-axis. Larger uncertainty of cell positions in the early stages indicates metastable state

The mini-cluster approximation allows us to apply GPseudoRank to larger datasets. Figure 6 shows that the uncertainty in the ordering for the Klein dataset (see Section 2.7.2) clearly exceeds that of other datasets in the early stages of the process. There is high uncertainty of cell positions at the beginning of the process as seen in the large area of intermediate densities in the lower left of Figure 6. This reflects the metastable state found early in the main branch in Figure 2c in Haghverdi *et al.* (2016). According to Haghverdi *et al.* (2016), such states can be defined as states with a high density in diffusion pseudotime, as many cells progress through this state slowly. With GPseudoRank we are able to identify such states in terms of the uncertainty of the posterior cell position in terms of rank time: this uncertainty is large if many cells are in a similar state and their ordering is more uncertain compared to phases where cells are more clearly separated by their progress. That is, uncertainty in rank time corresponds to metastable states.

The time to convergence at the 1.1-level for the Gelman-Rubin statistic for the Klein dataset was 6 min on a laptop. For details on computation times for all single-cell datasets analyzed, see Table 5 in the Supplementary Material.

### 3.4 Multi-modal structure of posterior distributions

MDS shows that posterior distributions of cell position vectors tend to be multimodal (see Fig. 1). To understand better what this complicated structure of the posterior positions means biologically, we applied GPseudoRank to a small dataset of only 18 cells (Shalek13), as the structure of the posterior distribution of the cell orderings is easier to understand with a smaller number of possible orderings. We performed MDS (Supplementary Fig. 13a), clustered the MDS projections into four clusters, and then computed, for the medoids of the four clusters, the antiviral score as for Figure 5. The result is shown in Supplementary Figure 3b. It shows that differences between different regions of the posterior distribution correspond to differences mainly in the second part of the orders, rather than the first. More precisely, the different regions of the posterior of the orders correspond to different trajectories of the antiviral score in the second part of the orders. While there is little uncertainty in the first half of the orders, the cells in the second half correspond to a metastable state, as in Figure 6. However, even in this metastable state, some orderings of cells are more likely than others as shown by the multi-modal structure of the posterior distribution. This indicates that there might be additional structure even in metastable states that can be revealed by algorithms such as GPseudoRank.

## 4 Discussion

GPseudoRank is a new type of Gaussian process latent variable model for pseudotemporal ordering. It samples orderings instead of pseudotimes, with combinatorial proposal moves designed to allow the Metropolis-Hastings sampler to make large changes to permutations and still achieve a high acceptance rate. This specific proposal distribution allows the sampler to explore complicated posterior distributions (see Fig. 1). Point estimation methods are only able to find a single estimate of the order, and are therefore at most able to capture one mode or find an estimate that lies between several modes (see again Fig. 1).

The applications to scRNA-seq and RT-PCR data illustrate another advantage of sampling from the posterior of orderings: the amount of uncertainty about the position of a cell can vary with time. In the Shalek dataset, the uncertainty is lowest in the middle of the process, where the heterogeneity of cells with regard to their progress through the response to the infection is highest. This identifies parts of the process with increased change and higher biological variability compared to technical noise. For other datasets, the noise levels are highest at the beginning (Klein data), in the middle (Stumpf data), or at the end (Shalek13 data).

The uncertainty of the orders is relevant to any further analysis that models scRNA-seq data in terms of time-series data. This applies, for instance, to any type of network inference where the order of the input time series is relevant, including GP models (Penfold *et al.*, 2012) and vector-autoregressive ones (Opgen-Rhein and Strimmer, 2007). Alternatively, identifying the regions of the process where the uncertainty of a cell’s position is low can support the selection of suitable cells for the clustering of genes for example.

Variational inference, which avoids sampling altogether, is considered a computationally efficient if only approximate Bayesian inference alternative to MCMC sampling. Considering that it samples from the full posterior distribution of the orders, GPseudoRank is very efficient and though runtimes obviously exceed those of well-designed variational methods (Ahmed *et al.*, 2018), the mini-cluster approximation allows GPseudoRank to be applied to large datasets without losing much insight concerning the structure of the posterior distribution. GPseudoRank with the mini-cluster approximation described in Section 2.6 takes 6 min to converge on a laptop for a dataset with more than 1500 cells. GPseudoRank can be applied to medium-sized datasets without approximation methods, taking about 50 min to converge at the 1.1-level of the Gelman-Rubin statistic for a dataset with 550 cells. However, with the mini-cluster approximation it takes 1 minute to reach the same level of convergence.

Overall, GPseudoRank offers new insights into biological phenomena and experimental artifacts. It quantifies the amount and variability of uncertainty in single-cell ordering (Figs 1, 3, and 6). Assessing the degree of uncertainty enables spotting experimental batch effects created by sampling from a continuous spectrum of developmental stages at only a few capture times. Our approach is also able to identify precocious cells (Fig. 4). By combining a geodesic pseudotime mapping with sampling permutations, GPseudoRank also avoids edge effects present in other GP methods for pseudotime ordering (Fig. 5).

Except for relative measurements like qPCR, GPseudoRank is applied to log-transformed data. This is a frequent procedure for many pseudotime methods: see among many others Haghverdi *et al.* (2016); Ji and Ji (2016); Reid and Wernisch (2016); Welch *et al.* (2016). Modelling count data directly in GPseudoRank could be achieved by a change in the likelihood function to, say, the negative binomial distribution with GPs modelling the mean. However, this would require additional sampling of latent mean values for a small gain in accuracy over a log normal approximation, which is usually very accurate for large count data.

We have illustrated GPseudoRank on a number of scRNA-seq datasets. Welch *et al.* (2017) developed a GP-based method for the inference of multi-omics pseudotime profiles through manifold alignment. A similar extension of GPseudoRank to the multi-omics case would allow insight into time-varying and multi-modal uncertainty structure of orderings for the multi-omics case.

Ordering problems are not restricted to the analysis of single-cell data. For instance, with clinical health record data the actual time of the onset of a disease is not usually known. It would be interesting to use an approach similar to GPseudoRank to order the measurements for different patients relative to each other. Unlike in the case of cells, the order and times of the measurements are known for each individual person. However, neither the rate of progression of the illness for the individual person, which is similar to the difference between actual time and pseudotime, nor the relative progression of the illness across different people are known. Generally, our approach of proposing local and wider proposal moves for MCMC in a sample space of distributions suggests new ways of addressing a number of discrete sampling problems, such as covariate selection or ranking in mixture models for clustering.

## Funding

M.S., J.R., and L.W. are funded by the UK Medical Research Council (Grant Ref MC_UU_00002/1).

*Conflict of Interest*: none declared.

## Supplementary Material

Supplementary Data
