# Supplementary material for: GPseudoRank: a permutation sampler for single cell orderings
Source: Bioinformatics. 2018 Jul 25;35(4):611–8. doi: 10.1093/bioinformatics/bty664 (PMC6230469; doi:10.1093/bioinformatics/bty664)
Supplement: Supplementary Data [file bty664_supplementary_materials.pdf]

GPseudoRank: supplementary materials

Magdalena E Strauß[1,*], John E Reid[1,2], and Lorenz Wernisch[1]

[1] *MRC Biostatistics Unit, University of Cambridge, Cambridge CB2 0SR, UK*
[2] *Alan Turing Institute, London NW1 2DB, UK*
[*] *Corresponding author: strauss.magda@gmail.com*

Contents

1 Detailed results of the simulation studies 1

2 Further description of data sets 11

3 Approximation for large data sets 11

4 Ordering data sets without capture times 14

5 Summaries and representations of posterior distributions of orders 15

6 Further description of proposal distribution for orders 17

7 Computation times 22

1 Detailed results of the simulation studies

We assessed the 15 possible combinations of our four moves according to the following:

- Whether or not all the chains for all the simulated data sets for a particular combination of moves have converged by the 10,000th thinned sample at the 1.1, 1.07, 1.05, and 1.02 levels for the Gelman-Rubin (GR) $\hat{R}$-statistic in terms of both the log-likelihood and the L^1-distances of the sampled cell positions the true reference positions 1:90.
- The average number of samples across the 16 simulated data sets until convergence at all the above levels, where we computed the $\hat{R}$-statistics in intervals of 20 for the thinned samples.
- The maximum number of samples over the 16 simulated data sets until convergence at all the above levels (note again that the $\hat{R}$-statistics are in intervals of 20).

Tables 1, 2, and 3 list the values of these criteria for each combination of moves for the three different scenarios explored in our simulation studies. For each criterion, the best performing combination is marked in magenta.

Figures 1, 2 and 3 illustrate the performance of the different combinations of moves in terms of the $\hat{R}$-statistic of the log-likelihood. Each line in the plots represents the $\hat{R}$-statistic corresponding to one of the 16 simulated data sets. Figures 4, 5 and 6 do the same for the $\hat{R}$ for the L^1-distances.

Table 1: **Simulation 1, GR statistics on log-likelihood and L^1-distances from reference positions.** For each criterion, the best performing combination of moves is marked in magenta.

**moves**	**1**	**2**	**3**	**4**	**1,2**	**1,3**	**1,4**	**2,3**	**2,4**	**3,4**	**1,2,3**	**1,2,4**	**1,3,4**	**2,3,4**	**1,2,3,4**
GR<1.01 at end, logLk	0.75	0.31	0.13	0.81	0.75	0.56	0.94	0.38	0.81	0.69	0.56	0.81	0.94	0.44	0.63
GR<1.01 at end, L1-dist	0.63	0.38	0	0.88	0.5	0.56	0.94	0.19	0.88	0.69	0.44	0.69	0.88	0.69	0.81
GR<1.02 at end, logLk	0.94	0.75	0.38	1	1	0.81	1	0.75	1	0.94	0.94	0.94	1	0.94	1
av. # thin. samp. to GR<1.02,logLk	NaN	NaN	NaN	1314	2014	NaN	1379	NaN	1705	NaN	NaN	NaN	1561	NaN	1619
max # thin. samp. to GR<1.02,logLk	NaN	NaN	NaN	2571	3651	NaN	7391	NaN	3511	NaN	NaN	NaN	3231	NaN	3051
GR<1.02 at end, L1-dist	0.94	0.81	0.31	1	0.94	1	1	0.56	1	0.94	0.94	1	1	0.94	1
av. # thin. samp. to GR<1.02,L1-dist	NaN	NaN	NaN	1240	NaN	3025	1759	NaN	2056	NaN	NaN	2051	1855	NaN	2230
max # thin. samp. to GR<1.02,L1-dist	NaN	NaN	NaN	2811	NaN	5731	4571	NaN	6051	NaN	NaN	5751	3631	NaN	5331
GR<1.05 at end, logLk	1	1	0.88	1	1	1	1	1	1	1	1	1	1	1	1
av. # thin. samp. to GR<1.05,logLk	676	1630	NaN	539	881	834	435	2011	837	772	1039	766	710	1349	1026
max # thin. samp. to GR<1.05,logLk	1131	4071	NaN	1571	1491	1691	991	5351	1591	1491	3511	1671	1491	3691	2771
GR<1.05 at end, L1-dist	1	1	0.94	1	1	1	1	1	1	1	1	1	1	1	1
av. # thin. samp. to GR<1.05,L1-dist	1212	1149	NaN	539	1056	1271	729	1312	889	1114	1080	906	817	777	1104
max # thin. samp. to GR<1.05,L1-dist	2891	2611	NaN	1611	2931	3011	1891	2191	2031	2451	1971	1991	1771	2191	2791
GR<1.07 at end, logLk	1	1	1	1	1	1	1	1	1	1	1	1	1	1	1
av. # thin. samp. to GR<1.07,logLk	557	585	2456	402	610	597	382	1369	482	660	820	610	574	946	565
max # thin. samp. to GR<1.07,logLk	1051	1311	5431	771	1111	1651	831	3331	851	1291	2211	1331	1291	2031	1491
GR<1.07 at end, L1-dist	1	1	0.94	1	1	1	1	1	1	1	1	1	1	1	1
av. # thin. samp. to GR<1.07,L1-dist	884	875	NaN	485	770	801	585	1056	625	639	804	486	605	632	716
max # thin. samp. to GR<1.07,L1-dist	2131	1831	NaN	1511	2211	1991	1351	1991	1391	1471	1771	1431	1071	2051	2631
GR<1.1 at end, logLk	1	1	1	1	1	1	1	1	1	1	1	1	1	1	1
av. # thin. samp. to GR<1.1,logLk	435	502	1864	246	377	376	287	812	330	500	521	475	449	605	396
max # thin. samp. to GR<1.1,logLk	851	1191	4171	471	911	931	611	2131	791	1051	1291	991	791	1231	1071
GR<1.1 at end, L1-dist	1	1	1	1	1	1	1	1	1	1	1	1	1	1	1
av. # thin. samp. to GR<1.1,L1-dist	571	620	1536	427	376	517	390	837	494	441	564	355	436	437	500
max # thin. samp. to GR<1.1,L1-dist	1851	1131	3451	1371	1031	951	1131	1591	911	1191	1311	1011	951	831	1051
av. AR	0.58	0.25	0.21	0.43	0.41	0.4	0.51	0.23	0.34	0.32	0.36	0.42	0.42	0.29	0.37
min. AR	0.52	0.22	0.19	0.38	0.39	0.37	0.45	0.21	0.32	0.3	0.34	0.39	0.39	0.27	0.35
max. AR	0.66	0.28	0.23	0.52	0.46	0.44	0.59	0.26	0.4	0.37	0.39	0.48	0.48	0.32	0.42

Table 2: **Simulation with fewer capture times.** For each criterion, the best performing combination of moves is marked in magenta.

**moves**	**1**	**2**	**3**	**4**	**1,2**	**1,3**	**1,4**	**2,3**	**2,4**	**3,4**	**1,2,3**	**1,2,4**	**1,3,4**	**2,3,4**	**1,2,3,4**
GR<1.01 at end, logLk	0	0.44	0.06	0	0.56	0.56	0	0.25	0.25	0.63	0.69	0.31	0.63	0.44	0.56
GR<1.01 at end, L1-dist	0	0.63	0.13	0	0.44	0.63	0	0.31	0.56	0.75	0.75	0.44	0.5	0.81	0.56
GR<1.02 at end, logLk	0	0.75	0.25	0	0.75	0.88	0	0.75	0.69	0.94	0.88	0.56	0.69	0.88	0.94
av. # thin. samp. to GR<1.02,logLk	NaN	NaN	NaN	NaN	NaN	NaN	NaN	NaN	NaN	NaN	NaN	NaN	NaN	NaN	NaN
max # thin. samp. to GR<1.02,logLk	NaN	NaN	NaN	NaN	NaN	NaN	NaN	NaN	NaN	NaN	NaN	NaN	NaN	NaN	NaN
GR<1.02 at end, L1-dist	0	0.81	0.44	0	0.75	0.94	0	0.69	0.63	0.88	0.94	0.56	0.69	1	0.88
av. # thin. samp. to GR<1.02,L1-dist	NaN	NaN	NaN	NaN	NaN	NaN	NaN	NaN	NaN	NaN	NaN	NaN	NaN	2515	NaN
max # thin. samp. to GR<1.02,L1-dist	NaN	NaN	NaN	NaN	NaN	NaN	NaN	NaN	NaN	NaN	NaN	NaN	NaN	4831	NaN
GR<1.05 at end, logLk	0	0.88	0.88	0	0.75	1	0	1	0.69	0.94	1	0.56	0.69	1	1
av. # thin. samp. to GR<1.05,logLk	NaN	NaN	NaN	NaN	NaN	1784	NaN	1667	NaN	NaN	1267	NaN	NaN	992	1002
max # thin. samp. to GR<1.05,logLk	NaN	NaN	NaN	NaN	NaN	7291	NaN	3591	NaN	NaN	2391	NaN	NaN	2771	1871
GR<1.05 at end, L1-dist	0	0.88	0.88	0	0.75	1	0	1	0.69	0.94	1	0.56	0.69	1	1
av. # thin. samp. to GR<1.05,L1-dist	NaN	NaN	NaN	NaN	NaN	1721	NaN	1856	NaN	NaN	1142	NaN	NaN	1224	1054
max # thin. samp. to GR<1.05,L1-dist	NaN	NaN	NaN	NaN	NaN	6071	NaN	3671	NaN	NaN	4331	NaN	NaN	2451	1971
GR<1.07 at end, logLk	0	0.88	0.94	0	0.75	1	0	1	0.69	0.94	1	0.56	0.69	1	1
av. # thin. samp. to GR<1.07,logLk	NaN	NaN	NaN	NaN	NaN	1549	NaN	1069	NaN	NaN	955	NaN	NaN	866	836
max # thin. samp. to GR<1.07,logLk	NaN	NaN	NaN	NaN	NaN	7251	NaN	3351	NaN	NaN	1551	NaN	NaN	1571	1751
GR<1.07 at end, L1-dist	0	0.88	0.94	0	0.75	1	0	1	0.69	0.94	1	0.56	0.69	1	1
av. # thin. samp. to GR<1.07,L1-dist	NaN	NaN	NaN	NaN	NaN	1594	NaN	1335	NaN	NaN	787	NaN	NaN	932	827
max # thin. samp. to GR<1.07,L1-dist	NaN	NaN	NaN	NaN	NaN	5431	NaN	3591	NaN	NaN	1531	NaN	NaN	2311	1311
GR<1.1 at end, logLk	0	0.88	1	0	0.75	1	0	1	0.69	0.94	1	0.56	0.69	1	1
av. # thin. samp. to GR<1.1,logLk	NaN	NaN	1637	NaN	NaN	1319	NaN	759	NaN	NaN	759	NaN	NaN	657	595
max # thin. samp. to GR<1.1,logLk	NaN	NaN	3211	NaN	NaN	7171	NaN	2491	NaN	NaN	1371	NaN	NaN	1291	1571
GR<1.1 at end, L1-dist	0	0.88	1	0	0.75	1	0	1	0.69	0.94	1	0.56	0.69	1	1
av. # thin. samp. to GR<1.1,L1-dist	NaN	NaN	1641	NaN	NaN	1157	NaN	871	NaN	NaN	729	NaN	NaN	767	679
max # thin. samp. to GR<1.1,L1-dist	NaN	NaN	3211	NaN	NaN	4871	NaN	1771	NaN	NaN	1331	NaN	NaN	1871	1171
av. AR	0.49	0.25	0.21	0.37	0.41	0.4	0.43	0.23	0.34	0.32	0.36	0.41	0.41	0.29	0.37
min. AR	0.38	0.22	0.19	0.3	0.34	0.37	0.34	0.21	0.3	0.3	0.34	0.35	0.31	0.27	0.35
max. AR	0.6	0.28	0.23	0.47	0.46	0.44	0.59	0.26	0.39	0.37	0.39	0.48	0.48	0.32	0.41

Table 3: **Simulation with higher noise levels.** For each criterion, the best performing combination of moves is marked in magenta.

**moves**	**1**	**2**	**3**	**4**	**1,2**	**1,3**	**1,4**	**2,3**	**2,4**	**3,4**	**1,2,3**	**1,2,4**	**1,3,4**	**2,3,4**	**1,2,3,4**
GR<1.01 at end, logLk	0.63	0.38	0	0.88	0.38	0.31	0.69	0.25	0.56	0.75	0.44	0.56	0.5	0.5	0.44
GR<1.01 at end, L1-dist	0.5	0.5	0	0.88	0.63	0.25	0.69	0.44	0.5	0.56	0.56	0.44	0.38	0.5	0.5
GR<1.02 at end, logLk	0.69	0.69	0.19	0.94	0.88	0.69	1	0.56	1	0.94	0.94	1	0.94	0.94	0.75
av. # thin. samp. to GR<1.02,logLk	NaN	NaN	NaN	NaN	NaN	NaN	1586	NaN	1816	NaN	NaN	2024	NaN	NaN	NaN
max # thin. samp. to GR<1.02,logLk	NaN	NaN	NaN	NaN	NaN	NaN	3191	NaN	5011	NaN	NaN	7351	NaN	NaN	NaN
GR<1.02 at end, L1-dist	0.88	0.88	0.13	0.94	0.81	0.63	0.94	0.81	0.94	1	0.75	0.94	0.94	0.88	0.88
av. # thin. samp. to GR<1.02,L1-dist	NaN	NaN	NaN	NaN	NaN	NaN	NaN	NaN	NaN	2596	NaN	NaN	NaN	NaN	NaN
max # thin. samp. to GR<1.02,L1-dist	NaN	NaN	NaN	NaN	NaN	NaN	NaN	NaN	NaN	5091	NaN	NaN	NaN	NaN	NaN
GR<1.05 at end, logLk	0.94	1	0.75	1	1	1	1	0.94	1	1	1	1	1	1	1
av. # thin. samp. to GR<1.05,logLk	NaN	2549	NaN	769	1150	1640	824	NaN	970	1340	1346	977	989	1572	1012
max # thin. samp. to GR<1.05,logLk	NaN	6651	NaN	1691	2031	9591	1571	NaN	2611	2791	2991	2191	2331	4631	2351
GR<1.05 at end, L1-dist	1	1	0.69	1	1	0.88	1	1	1	1	1	1	1	1	1
av. # thin. samp. to GR<1.05,L1-dist	1357	1780	NaN	875	1146	NaN	824	1759	974	1065	1216	1249	949	1430	1482
max # thin. samp. to GR<1.05,L1-dist	3271	3751	NaN	1791	2131	NaN	1931	3911	2131	2531	2091	3331	3651	3071	3011
GR<1.07 at end, logLk	1	1	0.94	1	1	1	1	1	1	1	1	1	1	1	1
av. # thin. samp. to GR<1.07,logLk	594	1266	NaN	579	856	1444	730	1515	525	791	892	700	811	1085	647
max # thin. samp. to GR<1.07,logLk	1391	3791	NaN	1511	1431	9291	1431	3111	1251	1691	1851	1691	1791	2091	1391
GR<1.07 at end, L1-dist	1	1	0.81	1	1	1	1	1	1	1	1	1	1	1	1
av. # thin. samp. to GR<1.07,L1-dist	1029	1289	NaN	661	901	1610	601	1135	637	835	971	844	582	1156	1161
max # thin. samp. to GR<1.07,L1-dist	2491	2951	NaN	1691	1631	8671	1731	2571	1511	1571	1591	1691	1091	2251	2431
GR<1.1 at end, logLk	1	1	0.94	1	1	1	1	1	1	1	1	1	1	1	1
av. # thin. samp. to GR<1.1,logLk	419	810	NaN	380	564	1154	531	1077	427	592	785	615	586	739	527
max # thin. samp. to GR<1.1,logLk	831	1571	NaN	691	1111	8591	1391	1851	1211	1291	1611	1631	1631	1331	1331
GR<1.1 at end, L1-dist	1	1	0.94	1	1	1	1	1	1	1	1	1	1	1	1
av. # thin. samp. to GR<1.1,L1-dist	689	857	NaN	512	676	1346	435	977	505	567	731	622	480	729	844
max # thin. samp. to GR<1.1,L1-dist	2311	1711	NaN	1011	1151	8051	891	2351	1451	971	1311	1511	891	1371	2291
av. AR	0.69	0.46	0.41	0.56	0.57	0.55	0.63	0.44	0.51	0.49	0.53	0.57	0.57	0.47	0.53
min. AR	0.66	0.42	0.37	0.54	0.54	0.52	0.6	0.39	0.48	0.46	0.49	0.54	0.54	0.44	0.5
max. AR	0.74	0.49	0.45	0.63	0.6	0.57	0.69	0.47	0.54	0.52	0.55	0.61	0.61	0.5	0.56

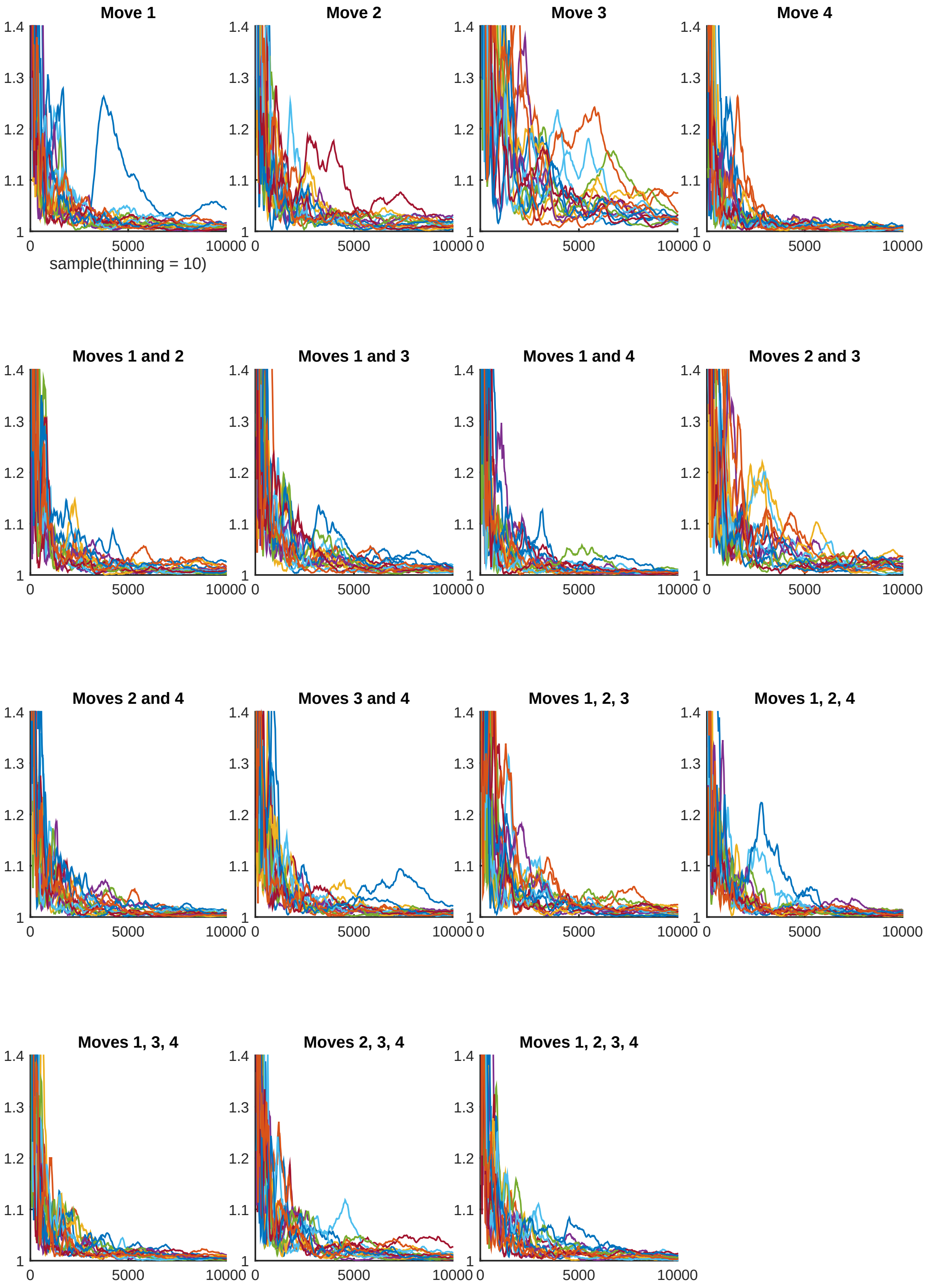

Figure 1: **Simulation 1: GR statistic for log-likelihood.** 16 simulated data sets with 5 MCMC chains per data set.

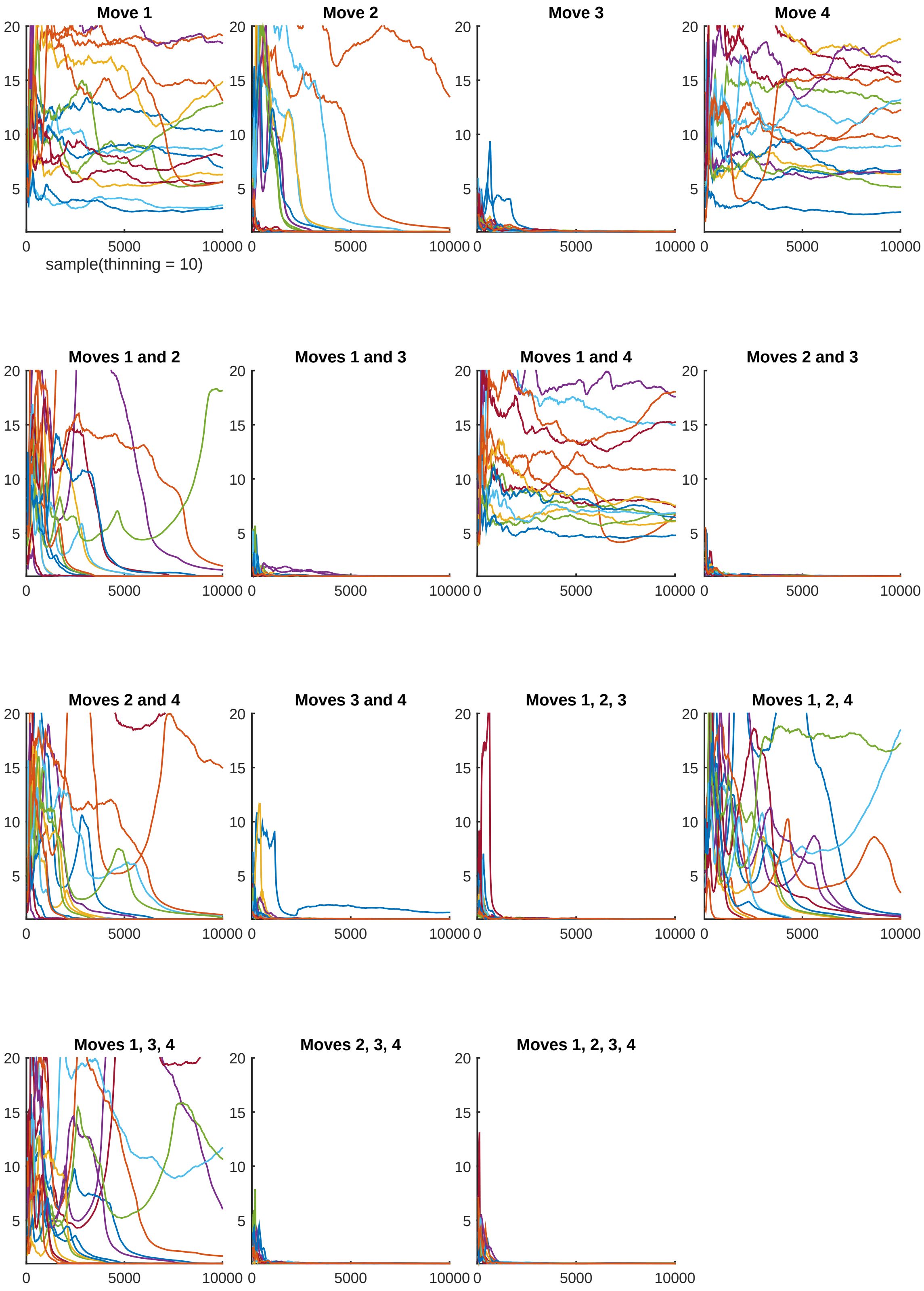

Figure 2: **Simulation with fewer capture times: GR statistic for log-likelihood.** 16 simulated data sets with 5 MCMC chains per data set.

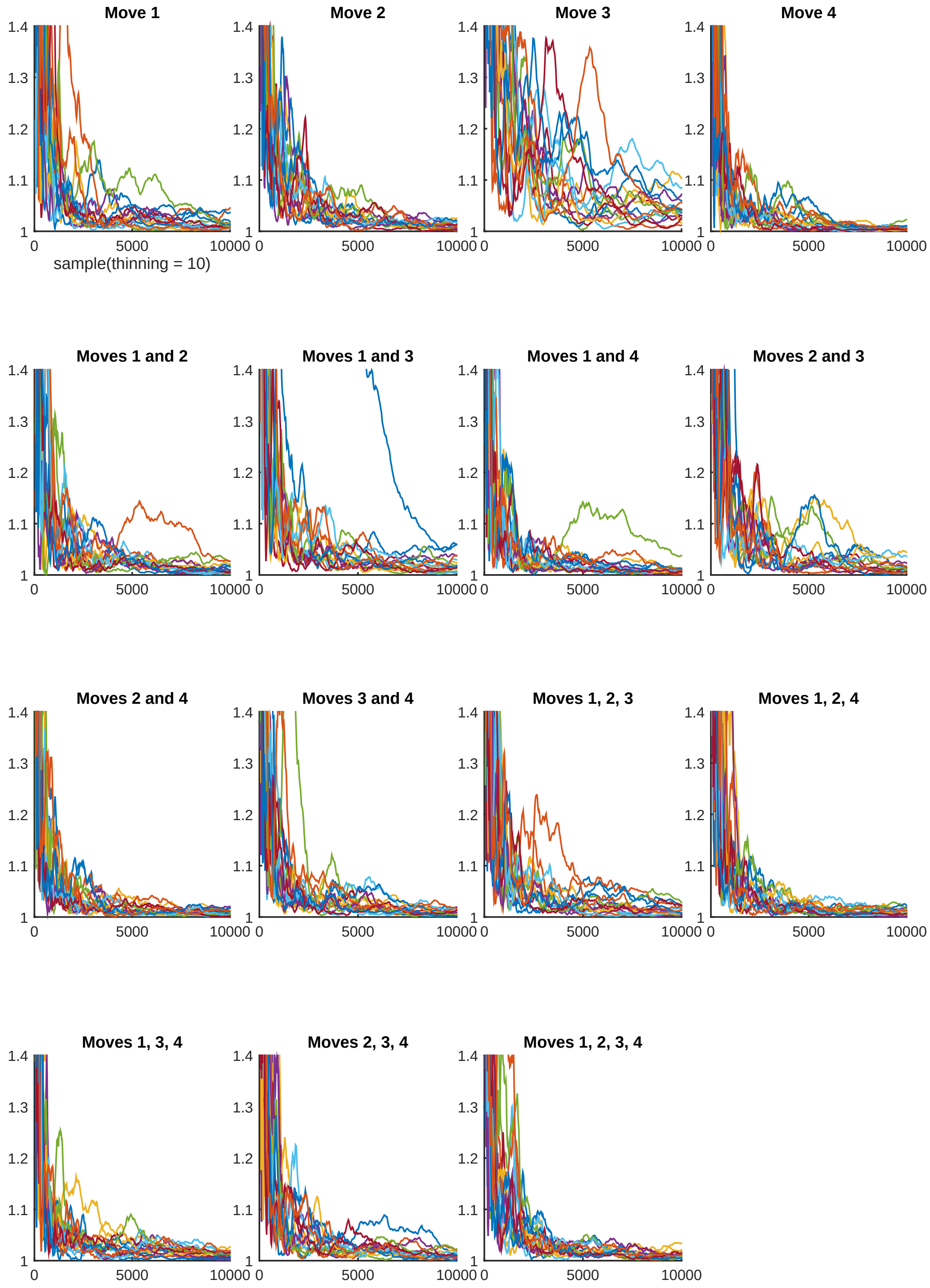

Figure 3: **Simulation with more noise: GR statistic for log-likelihood.** 16 simulated data sets with 5 MCMC chains per data set.

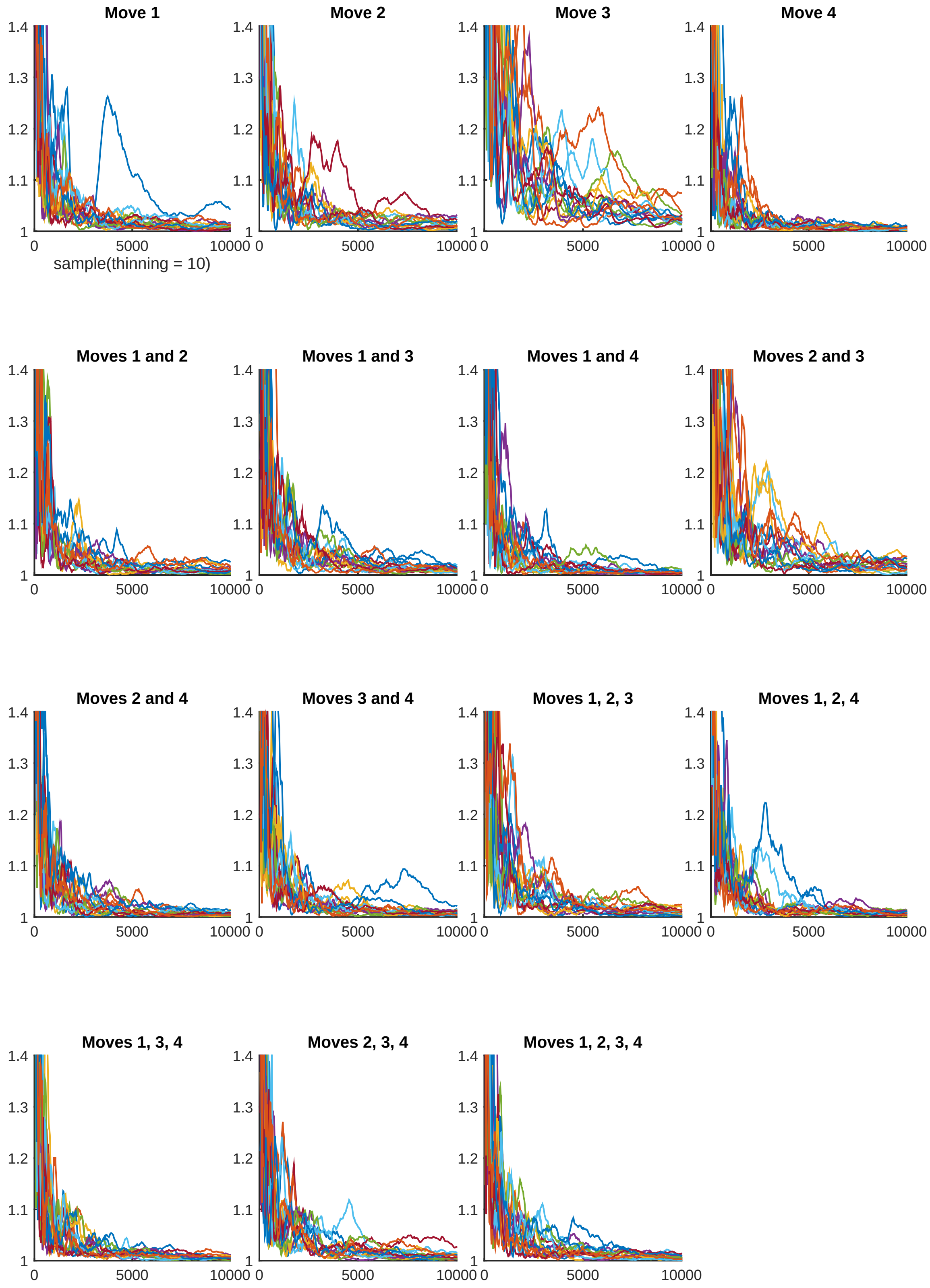

Figure 4: **Simulation 1: GR statistic for L^1-distance from ref. permutation.** 16 simulated data sets with 5 MCMC chains per data set.

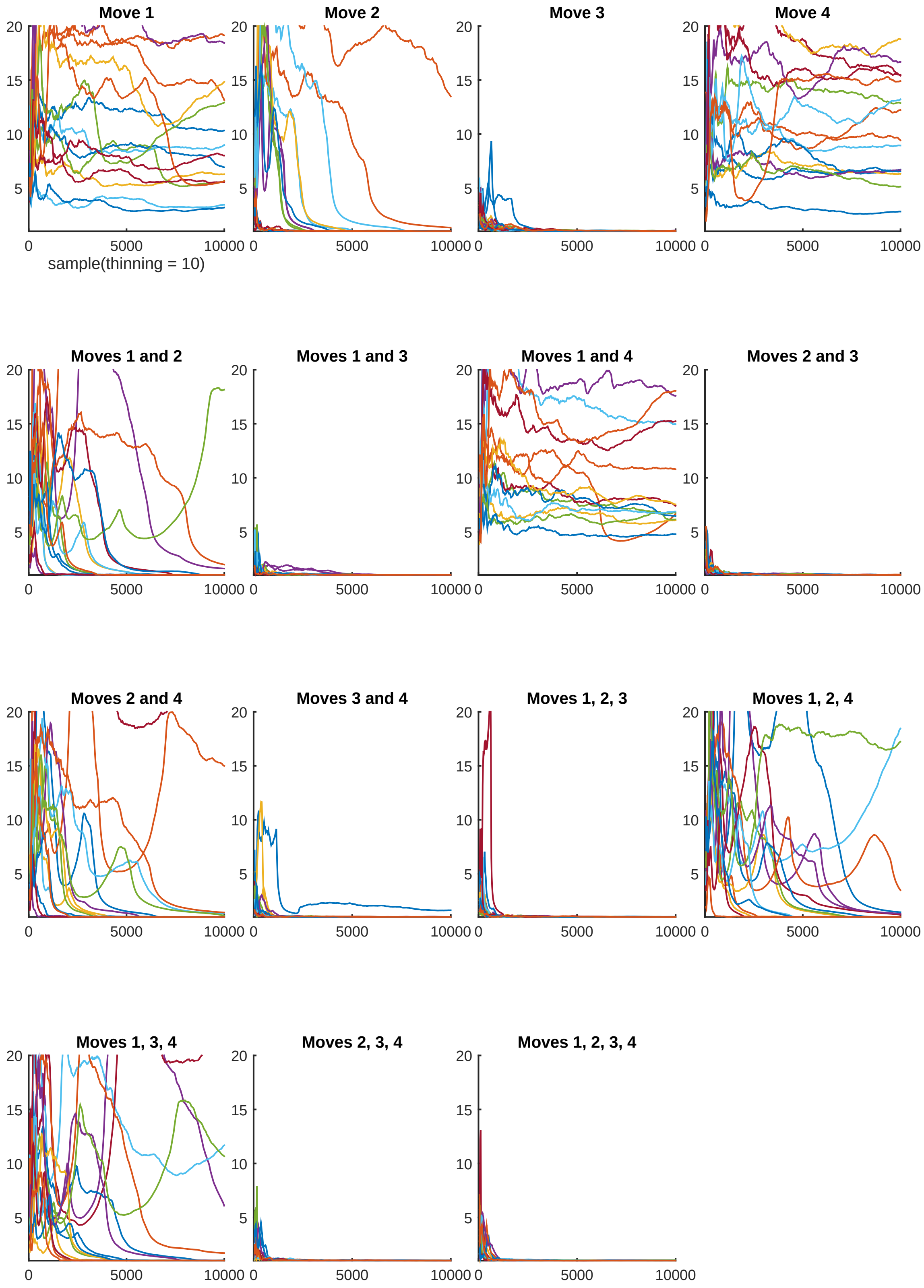

Figure 5: **Simulation with fewer capture times: GR statistic for $\mathbf{L^1}$-distance from ref. permutation.** 16 simulated data sets with 5 MCMC chains per data set.

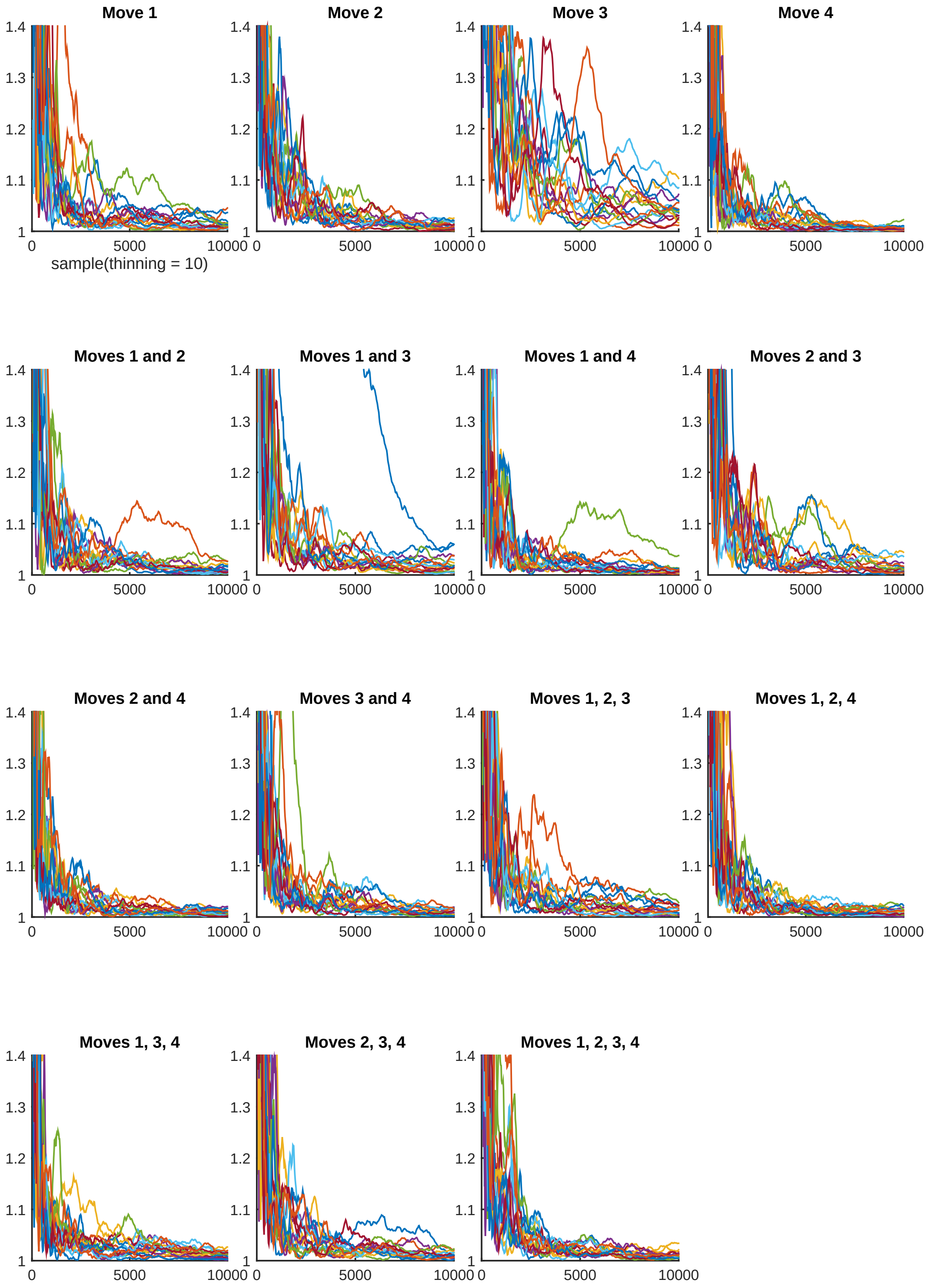

Figure 6: **Simulation with more noise: GR statistic for L^1-distance from ref. permutation.** 16 simulated data sets with 5 MCMC chains per data set.

2 Further description of data sets

We applied GPseudoRank to several publicly available single-cell expression data sets of different size and generated using different experimental protocols. The Shalek (Shalek *et al.*, 2014) data set is described in the main paper. GEO accession number: GSE48968.

Klein *et al.*, 2015 (Klein data set) used droplet barcoding to study RNA expression levels of mouse embryonic stem cells after Leukemia inhibition factor withdrawal. We use log-transformed data corrected for cell-cycle as in Haghverdi *et al.*, 2016, and apply GPseudoRank to the main branch identified in the latter publication, consisting of 1543 cells. Out of the genes used in Haghverdi *et al.*, 2016, we select 100 genes with high temporal variation according to an anova-test measuring differences between mean RNA expression levels (for each capture time, the mean is taken over all the cells at this capture time) for different capture times. The data, prior to performing the gene selection using anova, are available as supplementary material to Haghverdi *et al.*, 2016.

Shin *et al.*, 2015 (Shin data set) generated an in-vivo scRNA-seq data set of mouse adult hippocampal quiescent neural stem cells and their immediate progeny, using 101 of the cells captured for pseudotemporal ordering, excluding a small set of cells forming a separate branch and several outliers. In the absence of (at least two different) capture times, we choose the subset of genes to which to apply the GPseudoRank algorithm as follows: we first select all genes for which more than 70% of the cells have non-zero expression levels. Out of these genes we intersect the set of the 500 genes with the highest mean and that of the 500 genes with the highest variance, resulting in final set of 213 genes. We log-transformed the data before applying GPseudoClust. The data are available as supplementary material to Shin *et al.*, 2015.

Stumpf *et al.*, 2017 (Stumpf data set) generated an RT-PCR data set from two cell lines, following the development of mouse embryonic stem cells along the neuronal lineage. The data set consists of 96 genes including two loading controls, and 96 cells per capture time (0h, 24h, 48h, 72h, 96h, 120h, 172h). We compute the pseudotime trajectory for both cell lines jointly.

For the preprocessing of the data, we follow the steps performed in Stumpf *et al.*, 2017, resulting in a preprocessed data set with 550 cells. We apply GPseudoRank to all the genes, excluding the two loading controls (Actb and Gapdh). The data are available on Mendeley Data (http://dx.doi.org/10.17632/g2md5gbhz7.1).

Shalek *et al.*, 2013 (Shalek13 data set) obtained scRNA-seq data from mouse bone-marrow-derived dendritic cells after exposure to lipopolysaccharide, the same condition as studied, among others, in Shalek *et al.*, 2014. All 18 cells were captured 4h after initial exposure. The data set containing many zeros, we consider all genes expressed in at least 30% of the cells. Out of these we intersect the 1000 with the highest variance with the 1000 with the highest mean, where we chose 1000 instead of 500 because there is less overlap between the two groups compared to the Shin data. This results in a final 142 genes. GEO accession number: GSE41265.

3 Approximation for large data sets

Following the recommendations for the number of clusters in Section 2.6 of the main paper, we use 21 small clusters for each of the 5 capture times of the Shalek data set, resulting in a total of 105 mini-clusters, and 15 mini-clusters for each of the 7 capture times of the Stumpf data set, resulting again in a total of 105 mini-clusters. For the droplet-based Klein data set with a main branch of 1543 cells, we use $\frac{1}{8}$th of the cells of each capture time, with a minimum of 30 cells per capture time. This results in a data set with 226 mini-clusters. Figure 7 illustrates the performance of the mini-cluster approximation on the Stumpf data set and compares it to the full algorithm. Figures 7a and 7b and Table 5 illustrate the boost in computational efficiency obtained for this data set by the approximation thanks to the much faster convergence of the approximate method and the more efficient likelihood computations. Figure 7c shows that the mini-cluster method approximates well the posterior means of the pseudotimes of the cells. Comparing Figures 7d and 7e, we see that both the level and the changes of pseudotime uncertainty over the course of pseudotime are preserved by the approximation.

While we recommend the mini-cluster approximation for our MCMC algorithm, previously sparse GPs

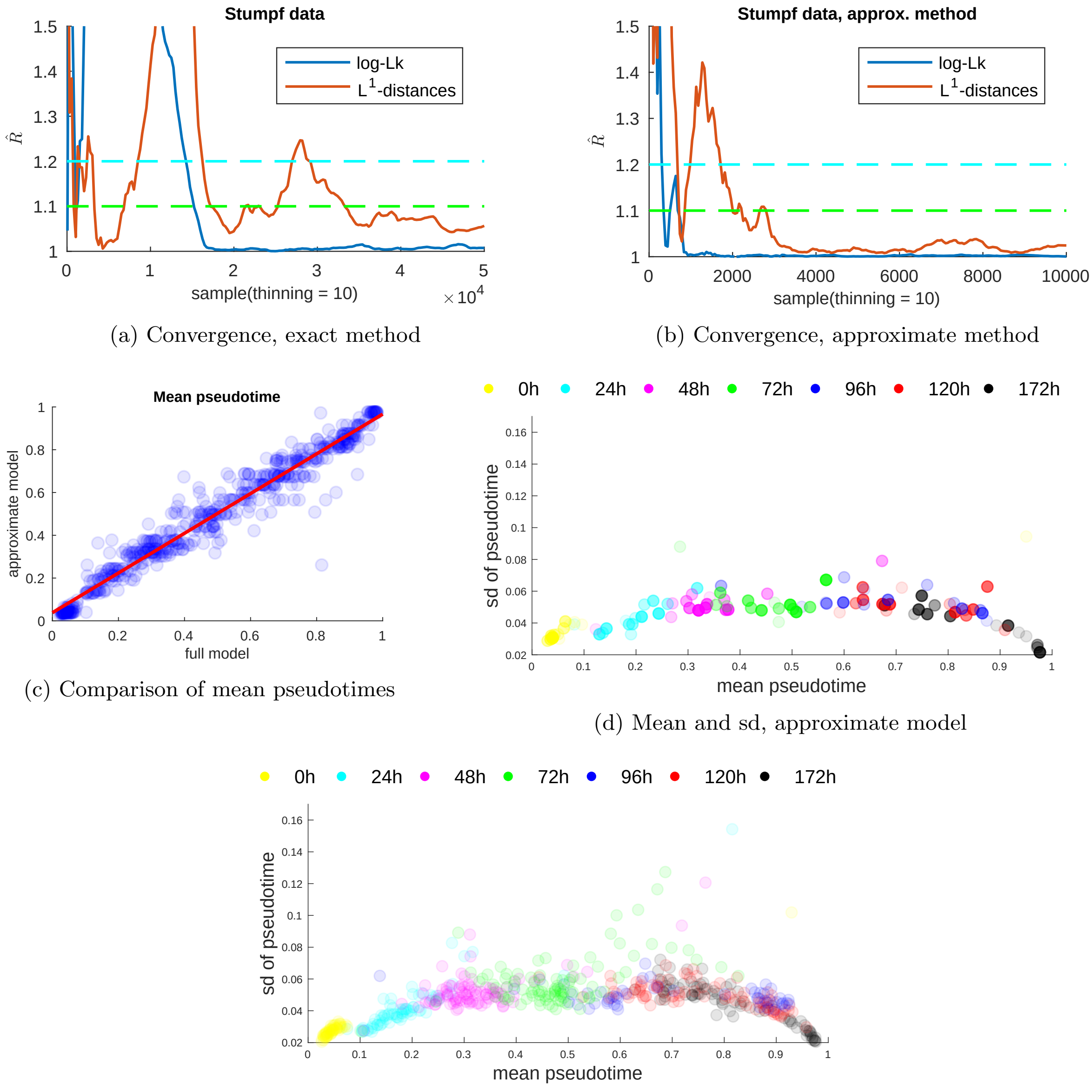

(a) Convergence, exact method

(b) Convergence, approximate method

(c) Comparison of mean pseudotimes

(d) Mean and sd, approximate model

(e) Mean and sd, full model

Figure 7: **Stumpf data, comparison between exact method and mini-cluster approximation.** a) $\hat{R}$-statistic for exact GPseudoRank inference. b) $\hat{R}$-statistic for approximation with mini-clusters. c) Scatterplot comparing the posterior means of the pseudotimes for each cell computed with the full model (x-axis) to those obtained using the mini-cluster approximation (y-axis). Semi-transparent dots show overlapping cells d) and e) Standard deviation of pseudotime as a function of mean pseudotime. Each dot corresponds to one cell. Semi-transparent dots show overlaps. d) approximate method, e) exact method.

have been used for GP latent variable models (Ahmed *et al.*, 2018; Reid and Wernisch, 2016). Sparse GPs approximate GPs in such a way that computations of inverses and determinants are only required for auxiliary GPs with a small number u of inducing points. While efficient alternatives with optimised inducing points have been implemented for variational inference of pseudotime (Ahmed *et al.*, 2018), optimisation at every iteration, or sampling the inducing points, would be computationally expensive in an MCMC sampler. We therefore use fixed inducing points and the sparse GP approximation of Snelson and Ghahramani, 2006, as in Reid and Wernisch, 2016. We obtain a method accurately estimating the posterior means of the pseudotimes (see Figure 8d), but overestimating the posterior uncertainty (see Figure 8f). The overestimation of uncertainty probably results from the fact that the sparse GP likelihood is less sensitive to changes of the ordering not crossing inducing points. Therefore, while providing accurate point estimates, sparse GPs might not be the ideal likelihood approximation for our

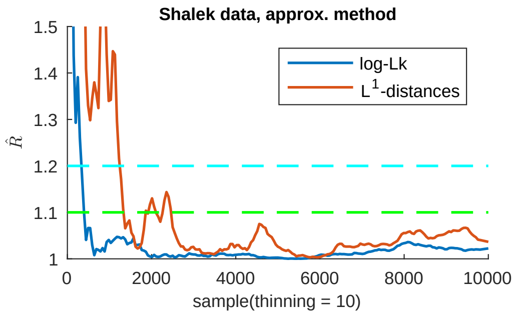

(a) Mini-cluster approximation: convergence

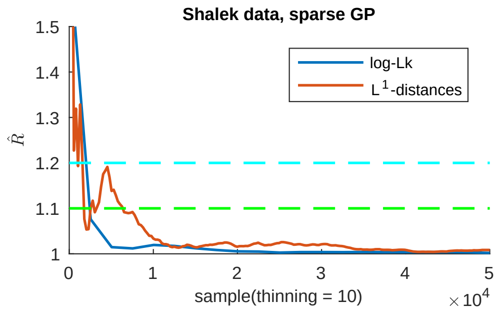

(b) Sparse GP: convergence

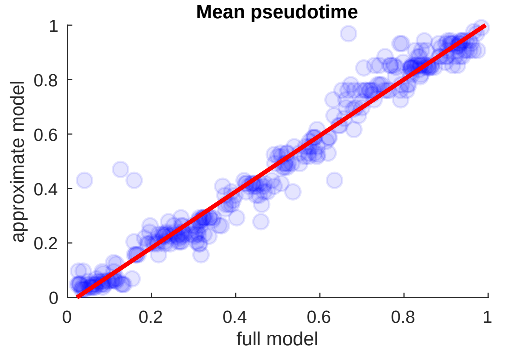

(c) Scatterplot comparing mean pseudotime between full model and mini-cluster approximation

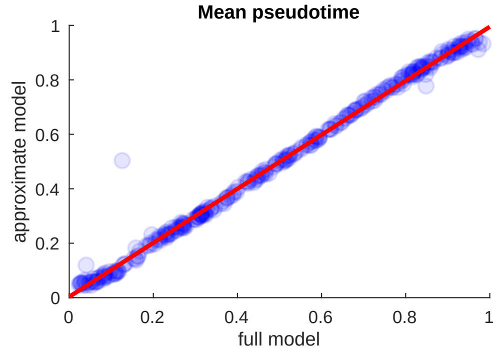

(d) Scatterplot comparing mean pseudotime between full model and sparse GP approximation

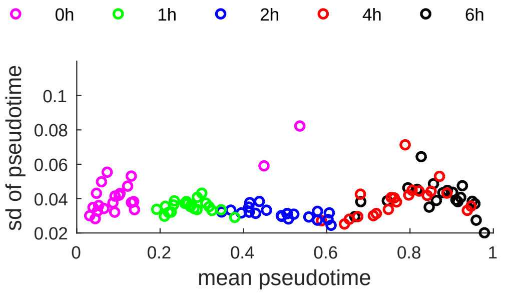

(e) Mean and sd of pseudotimes, mini-cluster approximation

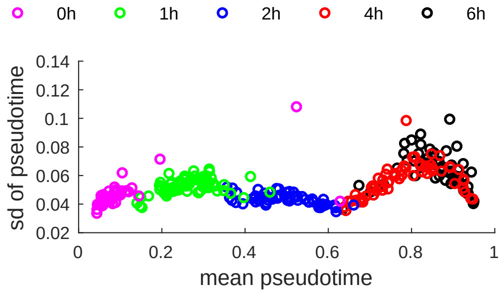

(f) Mean and sd. sparse GPs

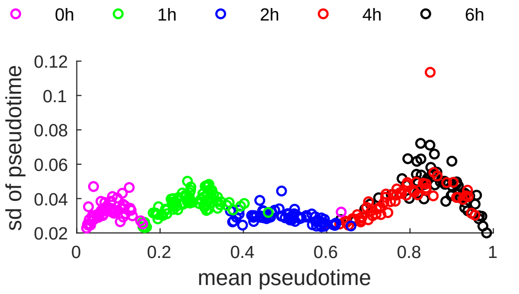

(g) Mean and sd., full model

Figure 8: **Shalek data, approximation methods.** a) and b) $\hat{R}$-statistic for approximation with mini-clusters (a) and sparse GPs (b). c) and d) Scatterplots comparing the posterior means of the pseudotimes for each cell computed with the full model (x-axis) to those obtained using the mini-cluster approximation (c) and sparse GPs (d). Semi-transparent dots show overlapping cells. e), f) and g) Standard deviation of pseudotime as a function of mean pseudotime, a) mini-cluster method, b) sparse GPs, c) full likelihood.

MCMC sampler. The mini-cluster approximation performs better at estimating the posterior uncertainty of orders and its change over the course of pseudotime.

4 Ordering data sets without capture times

While we showed convergence of our algorithm when the first two and the last two capture times for the Shalek data are merged for the initialisation in Figure 3 in the main paper, we now go further and show convergence with fully random starting paths, not using any capture time information. This means that a focus on large moves is required (for a discussion of local and larger moves, see Section 6); we use a combination of moves 2, 3, which are adaptive, and move 4, applying each of these moves with probability 0.3327, and move 5 with probability 0.0019. The settings for move 4 are the same as those used with capture time information, but we temper the proposals for moves 2 and 3 by a factor $\alpha = 0.1$, to make them larger. Figure 9a shows that, measured in terms of the $\hat{R}$ statistic, convergence is still good according to the recommendation to run chains until a threshold of 1.2 has been reached for the $\hat{R}$-statistic (see also Table 5). However, convergence is not reached as fast as when the capture times are used for the initialisation, compare Figure 9b to the corresponding figure for a chain initialised using some capture time information (Figure 3 in the main paper). We also plotted the Pearson correlations between capture times and posterior positions of the cells in the order (see Figure 9c, observing that both the posterior mean and median are highly correlated with the capture times, while the individual posterior samples are slightly less correlated. This illustrates the concept of uncertainty, where each sample will contain a number of less likely positions for a few individual cells, therefore being less correlated with the capture times than the posterior mean is. Therefore, it is interesting to see the two non-MCMC based methods in between the samples and the mean and median solutions by GPseudoRank.

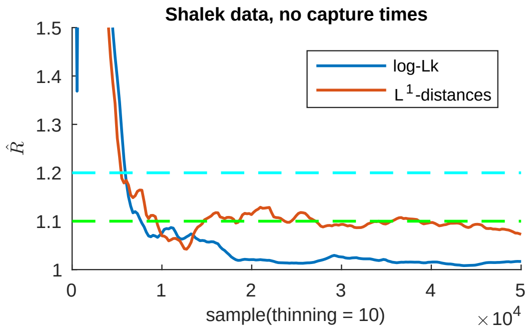

(a) $\hat{R}$**-statistic for the log-likelihood.** 12 chains initialised with fully random orders.

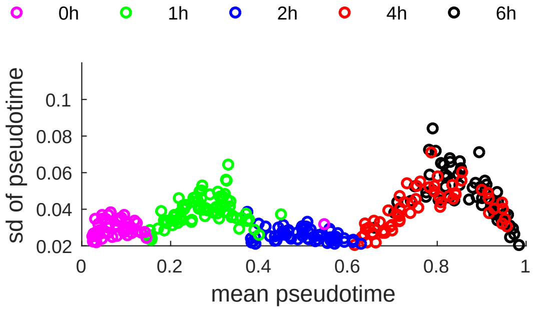

(b) **Uncertainty of pseudotime as a function of mean pseudotime.** For each cell, the mean pseudotime is plotted along the x-axis, and the respective standard deviation along the y-axis. Cells are coloured by capture time. The chain was initialised with a fully random order, discarding any information on capture times. Compare to Figure 3 in the main paper.

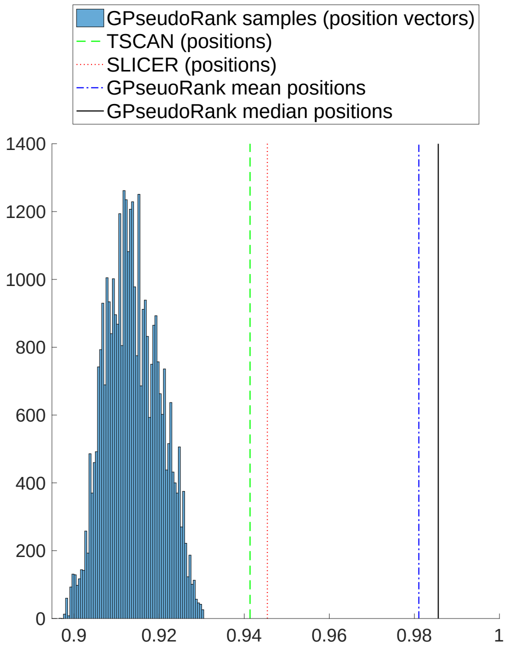

(c) **Pearson correlation to capture times.** Comparing the posterior samples of GPseudoRank, TSCAN, SLICER, mean positions inferred by GPseudoRank, median positions inferred by GPseudoRank.

Figure 9: **Shalek data, fully random initalisation without any use of capture time information.**

To illustrate further how GPseudoRank can be used for data sets with only one unique capture time for each cell, we applied it to the Shalek13 and Shin data sets (see Section 2 for a description of the data).

For the starting orders for the Shin data set, we fully randomly permuted the 101 cells. For the sampling

we applied moves 2, 3 and 4 with equal probabilities of 0.3327, and move 5, the reversal of the entire ordering, with a probability of 0.0019. The reason for not applying move 1, the swaps of neighbouring cells in the current order, is the fact that without capture time information for a not very small data set moves are preferred that propose larger changes to the ordering. Figure 10a illustrates the high level of convergence leading to practically similar posterior distributions of the positions in the order for each individual cell for 12 MCMC chains with fully random starting orders.

Without capture times, GPseudoRank samples the full symmetric distribution of orders, which includes an order and its reverse with equal probabilities (see Figure 10a). As there are no capture times, we cannot use positive or negative correlation with capture times to decide which of the two groups of orders need to be reversed, and therefore rely on different biological information. Figure 10b shows how to find which of the two groups of orders to reverse: both Sox11 and Eomes are markers for early intermediate progenitor cells (Ming and Song, 2011; Shin *et al.*, 2015), therefore those orders for which their expression level decreases need to be reversed.

With the Shalek13 data set, we proceed in a similar way concerning the reversal of orders. We use the same core antiviral score as in Figure 5 of the main paper. We reverse an order if the average for this score over the first 5 cells in the order is higher than that of the last 5 cells.

5 Summaries and representations of posterior distributions of orders

5.1 Distances from reference permutations

We use L^1-distances of cell position vectors, that is inverse permutations of orders. For instance, if the order of the cells is cell 4, cell 2, cell 1, cell 3, then the cell position vector is $(3, 2, 4, 1)$, that is cell 1 is at position 3, cell 2 is at position 2 etc. This distance measure is useful to obtain an overview over the posterior distribution of the orders, and it is the statistic we use, apart from the log-likelihood, for the assessment of convergence in terms of the Gelman-Rubin $\hat{R}$-statistic, see Figure 1 in the main text, Figures 4, 5, 6, 7a, 7b, 8a, 8b, and 9a in the supplementary materials, and Tables 1, 2, 3 and 5, also in the supplement. This distance statistic is particularly useful for convergence assessment because of its tendency to be comparatively close to a normal distribution (see Figures 11 and 12).

As the true order is not known unless in the case of applying the algorithm to microarray data for testing purposes and we do not want the reference permutation to depend on the data likelihood, we take a fixed (fixed for computing the distance of all samples from the posterior distribution for the same reference permutation), but randomly chosen permutation as the reference permutation. If there are capture times, we chose a reference permutation where cell positions are permuted only within the capture times.

While the tendency of the distribution of this distance statistic to have only one mode and be relatively close to a normal distribution makes it a good statistic for convergence assessment using the Gelman-Rubin statistic, it often fails to capture the multi-modal structure of the posterior distribution of the cell position vectors, and orders which are not particularly close to each other might still have similar distances from some reference permutations. This also makes the distance statistic an insufficient criterion for the comparison of different pseudotime methods (see Figures 11 and 12). For instance, the solutions obtained by TSCAN and SLICER look similar to each other with some of the reference permutations, although other reference permutations reveal the difference between the two solutions.

5.2 Multi-dimensional scaling and multi-modal structure of posterior distributions

To illustrate more in detail the complex structure of posterior distributions of orders, we also analysed the Shalek13 (Shalek *et al.*, 2013) data set, where the structure in the MDS projection of the vectors of cell positions is more clearly visible because of the overall smaller number of different possible orders due to the small size of the data set. Figure 13a shows the MDS projection. We performed k-medoids clustering with $k = 4$ on the projections, to identify groups of similar orders. For the orders corresponding to the medoids of the four groups we plotted the average expression levels of a set of antiviral genes also used

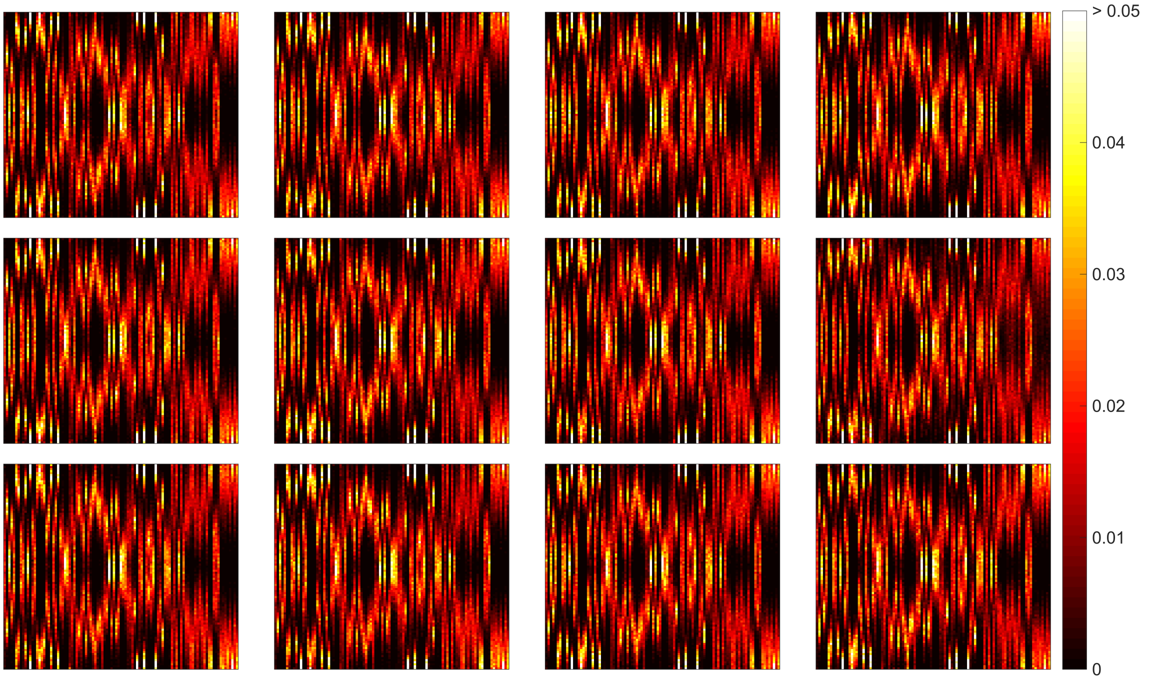

(a) **Distribution of cell positions.** Each matrix illustrates the posterior probabilities of the positions of the cells for one of 12 MCMC chains with different fully random starting orders. The cells are ordered along the horizontal axis in the same way for each of the subplots, the posterior density of the position of each cell in the order is plotted along the vertical axis. 15,000 thinned samples after burn-in of 15,000.

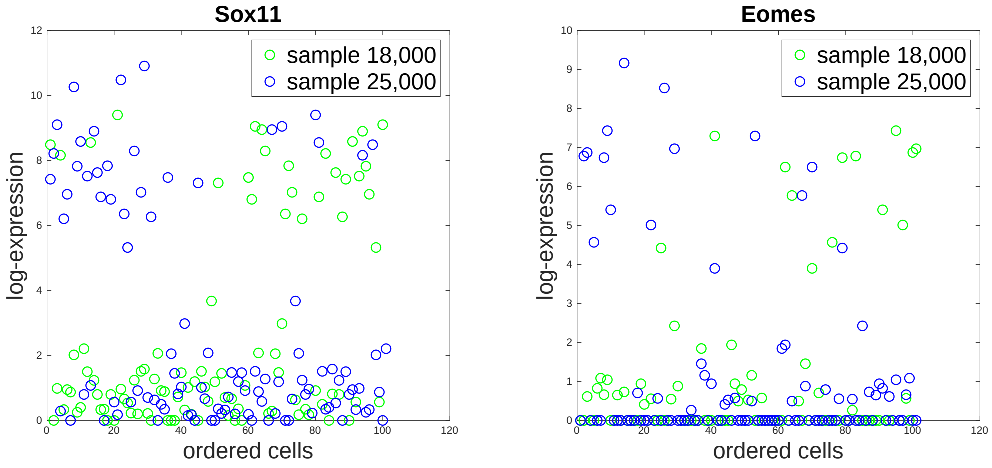

(b) **Marker genes.** The cells in green are ordered in terms of the 18,000th sample of the posterior distribution, the cells in blue in terms of the 25,000th sample.

Figure 10: **Analysis of Shin data.**

for the analysis of the other Shalek data set (Shalek *et al.*, 2014) in the main paper (see Section 3.2 and Figure 5 of the main text). Each subplot of Figure 13b compares the trajectory of the antiviral scores belonging to two different medoids in Figure 13a. We see that the beginning of the trajectory is very certain, while there is more uncertainty towards the end.

6 Further description of proposal distribution for orders

The MCMC moves described in Section 2.5 of the main paper are an efficient proposal distribution based on a combination of local and larger moves. The inclusion of larger moves, rather than just swapping neighbours in the ordering, is essential to avoid getting trapped in local maxima, in particular because the structure of the posterior distribution tends to be complicated, see Figure 2 in the main paper and Figure 13a in this text for examples.

The parameters n_0, n_3 and n_{3a}, γ, α_2 and α_3 are as in Section 2.5 of the main text.

1. Move 1 is a local move, in the sense that only pairs of neighbouring cells in the order are swapped.
2. Moves 2 and 3 are based on the observation that the acceptance rates of pairwise swaps of cells and reversals of orders between pairs of cells depend on the L^1-distances between these cells as elements in $\mathbb{R}^{n_g}$, where n_g is the number of genes. The reason for using L^1-distances rather than Euclidean ones is the lower sensitivity to outliers. We found a negative squared exponential function of the L^1-distances, that is a Gaussian kernel function, with a very slow decay an effective way of constructing the density for choosing pairs of cells for these moves. Moves 2 and 3 are adaptive, as once the chain has come closer to convergence, the cells close in L^1-distance are more likely to be closer to each other in the ordering.
3. Move 4 is similar to move 1, but less local, as it uses small random permutations of cells close to each other in the ordering rather than swaps of neighbours only.
4. Move 5 is accepted with probability 1, as it swaps around the entire ordering, therefore it does not change the likelihood of the GPseudoRank model. It is not absolutely necessary, but without it the distribution we sample on large data sets might not be symmetric, because of the lower probability of a swap of the entire ordering occurring with the other moves only, and we found this symmetry an additional help with the assessment of convergence without having to run multiple chains.

The number of possible permutations of T cells is equal to $T!$. This makes the choice of a suitable proposal distribution for the MCMC sampler a matter of importance. On the other hand, we want to provide a default parameter choice and a guide to setting the parameters depending on the size of the data set. However, every data set is different, therefore using non-default parameters for the proposal distribution may improve acceptance rates. Our general guidelines are the following:

It is generally most effective to use a combination of more local and larger moves, but the larger moves are crucial. When there is no capture time information a higher proportion of larger moves are required, and we recommend leaving out move 1 in that case. For the same reason, for using GPseudoRank on large data sets without the mini-cluster approximation, we recommend using moves 3, 4, and 5 only. For move 1 we found a recommended default of $n_0 = \lfloor nC/7 \rfloor$, where nC is the number of cells, to work well in general, for move 4, we generally set $n_3 = \lfloor nC/20 \rfloor$ and $n_{3a} = \lfloor nC/12 \rfloor$. However, n_3 and n_{3a} need to be decreased for large data sets. We generally recommend a flat distribution for choosing the cells for moves 2 and 3, with $\gamma = \frac{1}{8000}, \alpha_2 = 0.1$, and $\alpha_3 = 0.1$. However, the ideal setting depends on the specific data set and it is recommend to increase α_1 and α_2 and/or γ in case of low acceptance rates. Finally, for very small data sets, such as the Shalek13 data set, move 4 is similar to move 1 and move 2, and therefore it is sufficient to use moves 3 and 4. Table 4 lists in detail our parameter settings for the data sets analysed in this publication. We used a variety of different data sets with different experimental protocols and of different sizes (see Section 2) to provide good guidelines for the practitioner with regard to the parameter settings. The table shows that we sometimes deviated to some extent from the default parameter settings to optimise acceptance rates. However, default parameter settings tend to work fine in most cases, and a function to set default parameters is provided as part of the software package.

	$\frac{1}{\gamma}$	n_0	n_3	n_{3a}	α_2	α_3	moves
Shalek13	8000		3	3		0.01	3-5
Shin	1000		$\lfloor nC/20 \rfloor$	$\lfloor nC/12 \rfloor$	1	1	2-5
Shalek, CT	4000	$\lfloor nC/4 \rfloor$	$\lfloor nC/20 \rfloor$	$\lfloor nC/12 \rfloor$	1	1	1-5
Shalek, no CT	4000		$\lfloor nC/20 \rfloor$	$\lfloor nC/12 \rfloor$	0.1	0.1	2-5
Shalek, appr.	8000	$\lfloor nCl/7 \rfloor$	$\lfloor nCl/20 \rfloor$	$\lfloor nCl/12 \rfloor$	0.1	0.1	1-5
Stumpf	8000		$\lfloor nC/40 \rfloor$	$\lfloor nC/24 \rfloor$		0.1	3-5
Stumpf, appr.	8000	$\lfloor nCl/7 \rfloor$	$\lfloor nCl/20 \rfloor$	$\lfloor nCl/12 \rfloor$	0.1	0.1	1-5
Klein	8000	$\lfloor nCl/7 \rfloor$	$\lfloor nCl/20 \rfloor$	$\lfloor nCl/12 \rfloor$	0.1	0.1	1-5

Table 4: **Parameters used for proposal distributions for all scRNA-seq data sets.** nCl is the number of clusters used for the mini-cluster approximations. nC refers to the number of cells. CT refers to capture time, appr. to the mini-cluster approximation. Move 5 was used with probability 0.002, all other moves with equal probabilities. The Shalek data set was analysed twice, once using capture time information for the intialisation of the sampler (CT) and once without using this information (no CT).

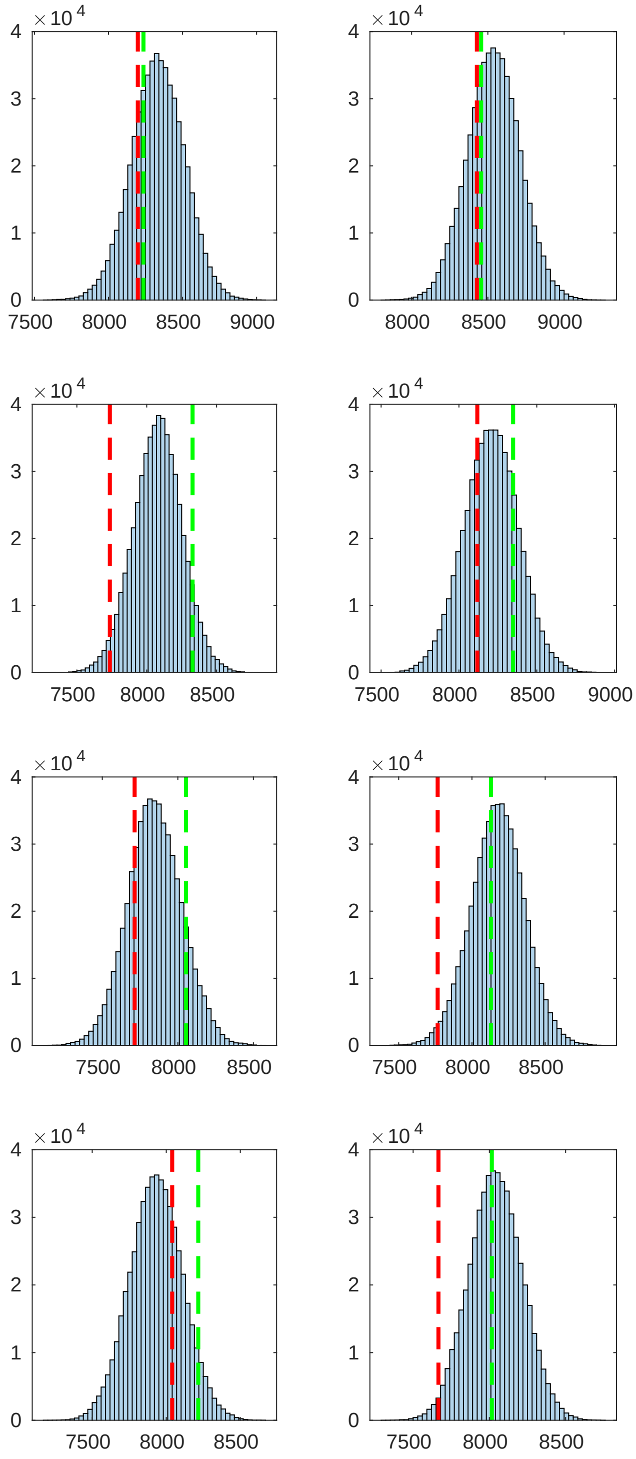

Figure 11: **L^1-distances from reference permutations.** For each subfigure, the reference permutation is a random permutation of the cells of each capture time. SLICER estimate represented as a red line, TSCAN as a green line.

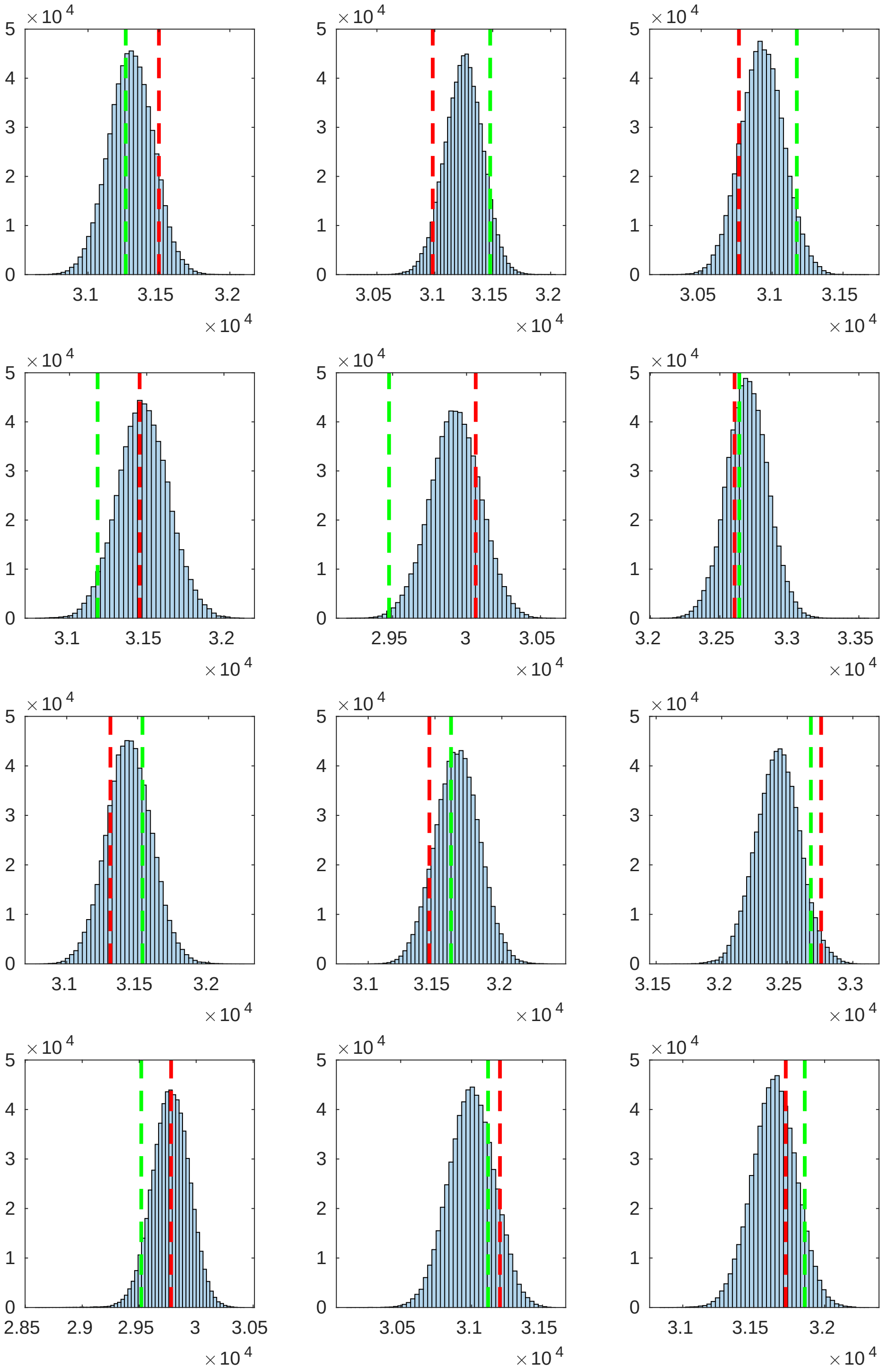

Figure 12: **$\mathbf{L^1}$-distances from reference permutations.** For each subfigure, the reference permutation is a random permutation of all the cells, not only within, but across capture times. SLICER estimate represented as a red line, TSCAN as a green line.

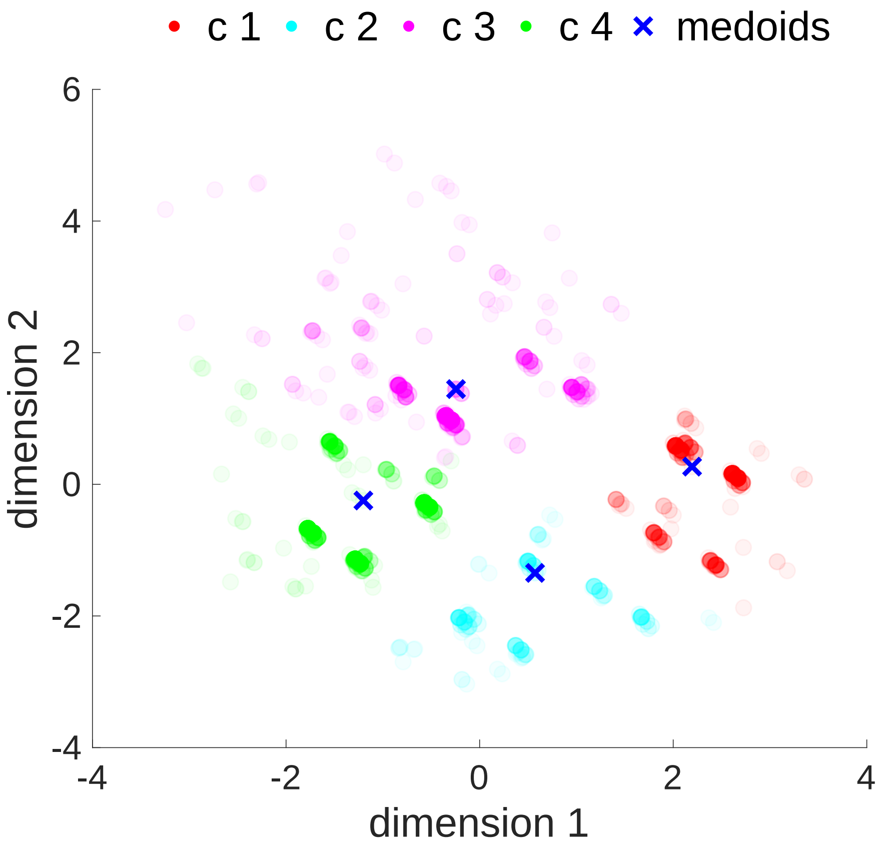

(a) MDS, 4 clusters.

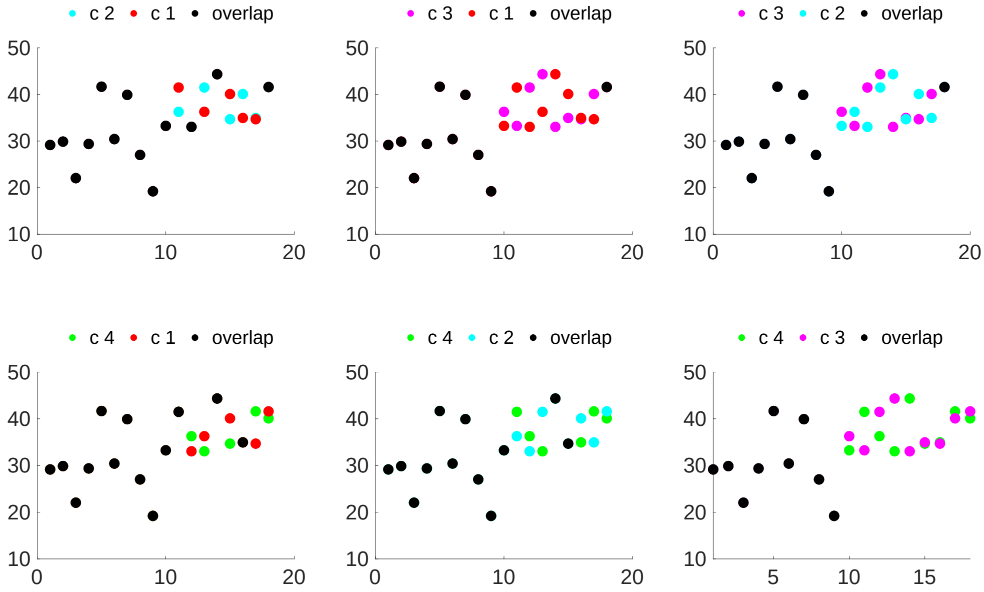

(b) Antiviral score for medoids, comparison.

Figure 13: **Analysis of Shalek13 data set.** a) MDS of posterior cell position vectors. Each posterior sample is represented by one semi-transparent dot. Samples with higher frequency, i.e. modes, therefore correspond to overlapping, less-transparent dots. Percentage of orders in clusters: c1: 21%, c2: 11%, c 3: 29%, c 4: 38% b) Antiviral score (as in Figure 5 of the main paper), each subplot compares ordered antiviral scores corresponding to one pair of medoids (as in a)).

7 Computation times

Table 5 lists the computation times on a laptop for all the single-cell data sets analysed. Computation times depend on the number of cells, the number of genes used for the analysis (this explain the slightly longer computation times per 1,000 samples for the Shin compared to the Shalek data with k-means approximation), and the number of samples required to reach convergence. For the number of cells and genes for each data set, see Section 2. As before, we assess convergence using the Gelman-Rubin statistic on the log-likelihood and the distances of the sampled cell position vectors from a reference permutation. The number of iterations until convergence listed in Table 5 is the number obtained from one random run of multiple chains. As the starting orders are random, different runs of the algorithm may lead to slightly different, but similar, numbers of iterations required.

	appr	cells	1,000 (thinned)	1.2 level	1.1 lev.	1.2 lev.	1.1 lev.
Shalek13	no	18	10 sec	250	550	3 sec	6 sec
Shin	no	101	35 sec	26,000	29,000	15 min	17 min
Shalek, CT	no	307	51 sec	3,750	6,000	192 sec	306 sec
Shalek, no CT	no	307	51 sec	5,500	22,450	281 sec	19 min
Shalek	yes	307	21 sec	1,300	1,350	27 sec	28 sec
Stumpf	no	550	159 sec	16,250	17,250	43 min	48 min
Stumpf	yes	550	25 sec	1,000	2,300	25 sec	58 sec
Klein	yes	1543	32 sec	9,300	11,300	5 min	6 min

Table 5: **Computation times until convergence for all single-cell data sets.** Convergence measured in terms of the $\hat{R}$-statistic on the log-likelihood and L^1-distances from a reference permutation. To demonstrate the efficiency of GPseudoRank and show that it can be run on a laptop, we performed the runtime analysis on a 2013 Macbook Pro with 2.3 GHz Intel Core i7 (4 cores) and 16GB DDR3, with matrix operations performed multi-threaded on all available cores. For the performance analysis the Matlab implementation of GPseudoRank was used. The table lists whether the full or approximate model were used (approx), how long it took to run 1,000 thinned samples (10,000 real samples), and how many samples and how much computing time were needed for convergence at the 1.2 and 1.1 levels for the $\hat{R}$-statistic for both the L^1-distances and log-likelihoods.

References

Ahmed, S. *et al.* (2018). GrandPrix: Scaling up the Bayesian GPLVM for single-cell data. *Bioinformatics*. bty533.

Haghverdi, L. *et al.* (2016). Diffusion pseudotime robustly reconstructs lineage branching. *Nat Meth*, **13**(10), 845–848.

Klein, A. *et al.* (2015). Droplet barcoding for single-cell transcriptomics applied to embryonic stem cells. *Cell*, **161**(5), 1187–1201.

Ming, G. and Song, H. (2011). Adult neurogenesis in the mammalian brain: Significant answers and significant questions. *Neuron*, **70**(4), 687–702.

Reid, J. E. and Wernisch, L. (2016). Pseudotime estimation: deconfounding single cell time series. *Bioinformatics*, **32**(19), 2973–2980.

Shalek, A. *et al.* (2013). Single-cell transcriptomics reveals bimodality in expression and splicing in immune cells. *Nature*, **498**, 236 EP –.

Shalek, A. K. *et al.* (2014). Single-cell RNA-seq reveals dynamic paracrine control of cellular variation. *Nature*, **510**, 363–369.

Shin, J. *et al.* (2015). Single-cell RNA-seq with Waterfall reveals molecular cascades underlying adult neurogenesis. *Cell Stem Cell*, **17**(3), 360–372.

Snelson, E. and Ghahramani, Z. (2006). Sparse Gaussian processes using pseudo-inputs. In Y. Weiss, P. Schölkopf, and J. Platt, editors, *Advances in Neural Information Processing Systems 18*, pages 1257–1264. MIT Press.

Stumpf, P. *et al.* (2017). Stem cell differentiation as a non-Markov stochastic process. *Cell Systems*, **5**(3), 268–282.e7.
